# Causal survival embeddings: Non-parametric counterfactual inference under right-censoring

**DOI:** 10.1177/09622802241311455

**Published:** 2025-02-11

**Authors:** Carlos García Meixide, Marcos Matabuena

**Affiliations:** 1 Instituto de Ciencias Matemáticas (ICMAT-CSIC), Madrid, Spain; 2 Departamento de Matemáticas, Universidad Autónoma de Madrid, Madrid, Spain; 3 27219 ETH Zürich, Zurich, Switzerland; 4 1812 Harvard University, Cambridge, MA, USA

**Keywords:** Causal inference, counterfactual distributions, survival analysis, right-censoring, reproducing kernel Hilbert spaces

## Abstract

Counterfactual inference at the distributional level presents new challenges with censored targets, especially in modern healthcare problems. To mitigate selection bias in this context, we exploit the intrinsic structure of reproducing kernel Hilbert spaces (RKHS) harnessing the notion of kernel mean embedding. This enables the development of a non-parametric estimator of counterfactual survival functions. We provide rigorous theoretical guarantees regarding consistency and convergence rates of our new estimator under general hypotheses related to smoothness of the underlying RKHS. We illustrate the practical viability of our methodology through extensive simulations and a relevant case study: The SPRINT trial. Our estimatort presents a distinct perspective compared to existing methods within the literature, which often rely on semi-parametric approaches and confront limitations in causal interpretations of model parameters.

## 1. Introduction

Estimating treatment effects using survival endpoints is a critical concern in the field of statistical methodology and translational research within the biomedical domain. However, the challenges related to obtaining data from randomized controlled trials hinder the establishment of robust experimental designs that aim to address questions related to treatment effects. Consequently, observational studies play a central role in medicine, and the development of new methods to accommodate the diverse sampling design problems that often arise is a major methodological concern. Personalized healthcare^1^ serves as an exemplary case in point, where planning tailored interventions is imperative to guide optimal treatments, and randomization may not be feasible due to ethical and resource constraints.

In the context of observational data, the cornerstone of statistical analysis revolves around the *potential outcomes framework*, which forms the foundation for causal assertions regarding treatment effects.^2–4^ As a general rule, we observe only one response per patient following a specific treatment arm, making it impossible to directly compare the effectiveness of multiple treatment strategies on the same individuals. This limitation leads to the need for inferential methods that can bridge this gap. In the field of counterfactual inference, the prevailing approach in the literature is based on the concept of average treatment effect (ATE). The ATE is a parameter of paramount importance as it quantifies the difference in means of potential outcomes distributions between arms,^5–7^ harmonizing with the principles of causal inference outlined by Pearl et al.^8^

A distinctive feature of survival analysis is that users are interested in modeling the conditional distribution of the response, rather than focusing solely on its specific characteristics, such as the conditional mean. This is because the conditional expectation often provides a limited picture of patients’ responses, given the significant variability and heterogeneity across individuals to a fixed therapeutic strategy.^9^ Furthermore, the presence of censoring complicates estimation and increases the bias of estimators, making the design of robust and efficient estimators a crucial task, given the limited availability of patient response data particularly when dealing with clinical treatments.

In practical terms, practitioners have widely employed estimators based on a hazard ratio. However, many of these strategies face challenges in terms of causal interpretation, as is the case with Cox’s^10^ model. This has sparked considerable debate regarding the advantages and real-world possibilities that these models offer in practice.^11–13^ There has also been a question about the need to develop new models that overcome these limitations while remaining interpretable. Furthermore, a poorly specified selection of semiparametric estimators can lead to clinical conclusions that are far removed from the reality of treatment effects.^14,15^

Chernozhukov et al.^16^ proposed seminal methodology for regressing counterfactual distributions in censoring-free contexts. Reciprocally, in survival analysis under randomization it would suffice to fit one Kaplan–Meier^17^ curve per arm. However, in observational studies (or randomized experiments with imperfect compliance), this type of analysis becomes challenging.^18^ In this line, distributional extensions of the ATE have been considered in the literature through multiple lenses. For example,^19^ considers a bootstrap strategy to test distributional treatment effects while^20^ base their work on the theory of reproducing kernel Hilbert spaces (RKHSs).

In this line, estimating potential outcomes distributions directly is straightforward in randomized settings but becomes challenging in observational studies due to distributional disparities between group characteristics.^10^ Our approach focuses directly on a parameter that directly captures the gap between potential outcomes on the scale of survival probabilities. Let ${\tilde{T}}_{1}$ and ${\tilde{T}}_{0}$ be the potential outcomes representing how much time would an individual survive if exposed to treatment or not, respectively. We consider the (functional) parameter measuring counterfactual survival probability gain across arms

$$P({\tilde{T}}^{1}>t)-P({\tilde{T}}^{0}>t),\;t>0$$

which holds a causally valid meaning and does not depend on non-collapsible parameters or restrictive assumptions.^11,21^

We embrace a framework for estimating counterfactual distributions under right-censoring, leveraging the power of inference within a RKHS. Inspired by classical econometric concepts in the absence of censoring,^16,22,23^ we offer a way to dissect the differences between observational distributions^24,25^ through disentangling the various components contributing to these discrepancies: whether they arise from treatment effectiveness, baseline characteristics, or a combination of both. It provides a structured mechanism for rigorously assessing the origins of observed disparities between treatment groups.

To delve into specifics, we introduce the concept of *Causal Survival Embeddings* (CSEs). While building upon prior work designed for non-censored data, our methodology goes a step further by not only estimating kernel embeddings of counterfactual distributions^20^ but also enhancing their interpretability in terms of survival functions. Our method introduces a fresh perspective for comprehending treatment effects in observational studies. They quantify the contributions of various factors and surmount the interpretation limitations often encountered with traditional distributional survival models, such as those seen in the Cox model.

From a practical standpoint, our estimators offer several advantages. They exhibit favorable statistical properties in terms of convergence rates. Additionally, they are versatile enough to accommodate diverse sources of information, having the potential to encompass techniques like functional data analysis and address the intricate structures prevalent in contemporary medical research.

### 1.1. Our results and contributions

We introduce a general non-parametric estimator under right-censoring of counterfactual survival functions based on statistical inference on RKHSs.

- Our estimation procedure is a model-free approach based on embedding counterfactual distributions in RKHSs. This extends the prior work of Muandet et al.^20^ to handle censored data-generation environments, being our proposal the first strategy in the literature that adjusts for confounding in non-parametric estimation of survival functions.
- Traditional non-parametric survival estimators like Beran’s^26^ typically rely on strong smoothness conditions such as differentiability of density functions. In general, our approach does not require these hypotheses (only mild conditions on the moments of the kernel function). This makes our method more flexible and applicable to a wider range of scenarios.
- Theoretically, we are able to provide asymptotic behavior guarantees for our estimator and compute its convergence rate by employing techniques from Empirical Process Theory. We utilize these techniques to deduce the Hadamard-differentiability of an operator that takes values in a RKHS. While the Functional Delta Method^27^ is widely known for its application to general operators in Banach spaces, the interplay between the geometry of RKHSs and von Mises calculus remains relatively unexplored in the literature, with little research delving into this aspect.^28,29^
- Our procedure is computationally efficient since the main challenge lies in the estimation of conditional mean embeddings, a process that traditionally requires non-severe computational resources. Furthermore, our approach exclusively uses Kaplan–Meier weights, which are implemented in a highly efficient manner in widely recognized software packages, such as R’s *survival*.
- In addition, the framework of kernel embeddings allows for a natural reconstruction of probability measures from information encoded in elements belonging to Hilbert spaces of functions.^30^ The Supplemental material includes a brief explanation of how to recover counterfactual distributions from estimators of their kernel mean embedding. We display the potential and interpretability of our new estimator through its application to a pioneer analysis of the SPRINT trial,^31^ a celebrated governmental medical study tackling cardiovascular morbidity and mortality in the United States.
- We demonstrate that CSEs outperform existing and well-known methods in the literature, as showcased by simulations in Section 5.1. Specifically, our method surpasses the Adjusted Kaplan–Meier Estimator (AKME)^32^ and Causal Survival Forests.^33^

### 1.2. Related work

Different methods exist for treatment effect estimation in presence of censoring. A general procedure that can be found across the literature consists of the following steps. First, the ATE is causally identified without censoring and then a so-called *Censoring Unbiased Transformation*^34,35^ is used to create a pseudopopulation from the observed data in which the conditional mean survival time is the same as in the uncensored population. Second, methodology from semiparametric inference adapts the estimators to the censoring mechanisms.^36^ Alternative estimators of treatment effect include standardizing expected outcomes to a given distribution of the confounders (Robins’ g methods^37^), inverse probability of treatment weighting (IPTW) estimators^38,39^ and doubly robust estimators,^40^ which combine the two latter lines. In Xue et al.,^41^ the authors balance the covariates over an RKHS to avoid directly modeling the propensity score for estimating causal effects.

The utilization of tools from the RKHS framework for right-censored data is relatively limited, with a primary focus on hypothesis testing,^42–44^ albeit outside the context of counterfactual inference. There have also been efforts to perform hypothesis testing using RKHS’s in other incomplete information schemes such as missing reponses.^45^

### 1.3. Organisation of the paper

The paper is structured as follows. In Section 2 we rigorously introduce the formal elements that constitute the basis of our work by specifying: The fundamental random variables playing a role, which of them are observable and which are not, how they interact between them to generate incomplete information, and notation for their distribution functions. A self-contained description of the parameters of interest is presented next, accompanied by an opening introducing the notion of counterfactual distributions in survival analysis. Then we define their counterparts in a Hilbert space, leading to the notion of counterfactual mean embedding. Naturally, in Section 3 we develop the estimation theory that is needed in our setting, involving M-estimation on a space of functions that themselves take values in another space of functions. The asymptotic properties of our proposal estimator are investigated in Section 4, starting with preliminary definitions needed for its formalization followed by sufficient conditions for consistency and a convergence rate of non-parametric counterfactual inference under censoring. Detailed and extensive simulation studies can be found in Section 5, being Section 6 concerned with the workflow of our proposal in medical contexts, illustrating the usefulness of our methodology in a real application case related to cardiology. Finally, Section 7 closes the document with a discussion on the consequences of relaxing the censoring assumptions and other concerns regarding open directions.

## 2. Population elements

### 2.1. Setup

We start with a collection of random variables in the potential outcomes framework:^46^ ${\tilde{T}}^{0},{\tilde{T}}^{1}\in (0,+\infty )$ are potential outcomes of survival times of interest; ${\tilde{C}}^{0},{\tilde{C}}^{1}\in (0,+\infty )$ are potential outcomes of censoring times; $X\in R^{p}$ are individual vectors of covariates, p≥1 ; and Z∈{0,1} are individual treatment assignment indicators, where Z=0 denotes the control group and Z=1 denotes the treatment group. Next, we define the *realized* survival and censoring times respectively as

$$T=(1-Z){\tilde{T}}^{0}+Z{\tilde{T}}^{1},\;C=(1-Z){\tilde{C}}^{0}+Z{\tilde{C}}^{1}$$

The *observed* response is therefore $T^{*}:=\min\{T,C\}$

We define $T^{0}$ and $C^{0}$ as random variables distributed according to $F_{T^{0}}:=F_{T|Z=0}=F_{{\tilde{T}}^{0}|Z=0}$ , where $F_{V}$ denotes the distribution function of each random variable V . This function is mathematically relevant because we will see that conditional distributions of times coincide with conditional distribution of counterfactual times. $X^{z}$ is defined as the real p -dimensional random variable that follows the distribution of X conditional on Z=z , for z∈{0,1} . The event indicator is $\Delta =(1-Z)1({\tilde{T}}^{0}\leq {\tilde{C}}^{0})+Z1({\tilde{T}}^{1}\leq {\tilde{C}}^{1})$ . Additionally, it is worth noting that

$$\begin{array}{rl} & (1-Z)\min\{{\tilde{T}}^{0},{\tilde{C}}^{0}\}+Z\min\{{\tilde{T}}^{1},{\tilde{C}}^{1}\}\\ & \;=\min\{(1-Z){\tilde{T}}^{0}+Z{\tilde{T}}^{1},(1-Z){\tilde{C}}^{0}+Z{\tilde{C}}^{1}\}=\min\{T,C\} \end{array}$$

which could be interpreted as commutativity between censoring and realizing.

In practice, we observe an i.i.d sample $\left\{(T_{i}^{*},\Delta_{i},Z_{i},X_{i}){\}}_{i=1}^{n}∼(T^{*},\Delta ,Z,X\right)$ , which are draws containing incomplete information about the original random variables. In the following, $S_{V}=1-F_{V}$ denotes survival function of variable V in all cases.

### 2.2. Counterfactual survival functions

A key consideration for understanding counterfactual inference is that $S_{{\tilde{T}}^{1}|Z=1}=S_{Z{\tilde{T}}^{1}+(1-Z){\tilde{T}}^{1}|Z=1}=S_{T|Z=1}=:S_{T^{1}}$ but $S_{{\tilde{T}}^{1}|Z=1}\neq S_{{\tilde{T}}^{1}}$ because ${\tilde{T}}^{0}$ and ${\tilde{T}}^{1}$ may be dependent of Z . In other words ${\tilde{T}}_{1}$ and ${\tilde{T}}_{0}$ differ from the arm-conditional outcomes, which are observed values given an specific value of Z . To guarantee the identifiability of causal effects from observational data we have to respect the assumption that the potential outcomes are dependent of the treatment only via the covariates; i.e., there is no hidden confounding. This hypothesis is known as *unconfoundedness* or *ignorability*, which is a common hypothesis in observational studies. Throughout the paper, we term this assumption *conditional exogeneity*, that can be formally expressed as

$${\tilde{T}}^{0},{\tilde{T}}^{1}⊥⊥Z|X\text{ and }{\tilde{C}}^{0},{\tilde{C}}^{1}⊥⊥Z|X$$

Survival functions of potential outcome times conditional on the treatment indicator are of interest because of their involvement in an expression that aims to break down the difference between both *realized* distribution functions for Z=0,1 . This decomposition serves as a motivation for the foundational work of Chernozhukov et al.^16^ on counterfactual distributions. The decoupling is expressed as:

(1) $$\begin{array}{rl} S_{T^{1}}(t)-S_{T^{0}}(t)(t) & =F_{{\tilde{T}}^{0}|Z=0}(t)-F_{{\tilde{T}}^{1}|Z=1}(t)\\ & =\underset{\left(A\right)}{\underbrace{F_{{\tilde{T}}^{0}|Z=0}(t)-F_{{\tilde{T}}^{0}|Z=1}(t)}}+\underset{\left(B\right)}{\underbrace{F_{{\tilde{T}}^{0}|Z=1}(t)-F_{{\tilde{T}}^{1}|Z=1}(t)}} \end{array}$$

The equation comprises two terms, (A) and (B), which represent the distributional effect of covariate distributions and the distributional treatment effect on the treated, respectively. The difference between the realized outcome distributions can be attributed to either or both of these terms, and their estimation is valuable in understanding the origin of the difference in observed outcome distributions. If (A) is small with respect to (B), the difference between the observed outcome distributions is caused by the distributional difference on the treated (B). In the reciprocal configuration, the difference between the observed outcome distributions is due to (A), which is caused by distributional differences between the covariates in each group and not by the effects of the treatment. In the econometrics jargon, (A) quantifies a composition effect due to differences in characteristics and (B) stands for differences in the response structure.^16^

It is important to note again that $S_{T^{1}}(t)-S_{T^{0}}(t)\neq S_{{\tilde{T}}^{1}}(t)-S_{{\tilde{T}}^{0}}(t)$ . The latter accounts for the effects of treatments 0 and 1, but our approach delves into what is driving the first one to be non-zero. Observed outcome distributions are biased approximations to potential outcome distributions if the treatment assignment is not randomized (i.e. if X and Z are not independent).

We now see how these distributions related to potential outcomes that appear in distributional causal effects are related to the notion of counterfactual distribution, defined below. In the following, $F_{T^{0}|X^{0}=x}(\cdot )$ and $F_{T^{1}|X^{1}=x}(\cdot )$ are the conditional distribution functions that describe the stochastic assignment of survival times to people with characteristics x conditional on Z=0 or Z=1 , respectively. We indistinctly use the relation S=1−F . We need to introduce the notion of counterfactual distribution, which can be conceptualized as arising from either an alteration in the distribution of a set of covariates, which determine the outcome variable of interest; or as a modification in the association between the covariates and the outcome.

Definition 1 **Counterfactual distributions, Chernozhukov et al.^16^**

Whenever support $\left(F_{X^{1}}\right)$ ⊆ support $\left(F_{X^{0}}\right)$ ,

$$F_{T\langle 0|1\rangle }(\cdot ):=\int F_{T^{0}|X^{0}=x}(\cdot )dF_{X^{1}}(x)$$

An important result in Muandet et al.^20^ establishes a link between arm-conditioned and counterfactual distributions. It asserts that, in general, $S_{T\langle 0|0\rangle }=S_{{\tilde{T}}^{0}|Z=0}\text{ and }S_{T\langle 1|1\rangle }=S_{{\tilde{T}}^{1}|Z=1}$ . Moreover, if conditional exogeneity holds and support $(S_{X^{1}})=$ support $\left(S_{X^{0}}\right)$ then we also have $S_{T\langle 0|1\rangle }=S_{{\tilde{T}}^{0}|Z=1}\text{ and }S_{T\langle 1|0\rangle }=S_{{\tilde{T}}^{1}|Z=0}$

If assumptions of Lemma 1 are fulfilled, then term (A) in (1) satisfies

$$\begin{array}{rl} \left(A\right) & =S_{T\langle 0|1\rangle }(t)-S_{T\langle 0|0\rangle }(t)\\ & =\int F_{T^{0}|X^{0}=x}(t)dF_{X^{0}}(x)-\int F_{T^{0}|X^{0}=x}(t)dF_{X^{1}}(x) \end{array}$$

This makes clear that (A) is due to a shift between the covariate distributions $F_{X^{0}}$ and $F_{X^{1}}$ , as the only discrepancy between both integrals in the right hand side is originated by the measures. Meanwhile, as explained, (B) would quantify a treatment effect conditional to the intensive treatment arm.

### 2.3. Kernel embeddings

Let l:(0,+∞)×(0,+∞)→R be a kernel—symmetric positive semidefinite function—and H its associated RKHS.^47^ For generality purposes, we denote by X the covariate space. We assume conditional exogeneity and that the random variable $T^{0}$ satisfies the integrability condition $\int_{0}^{\infty }\sqrt{l(t,t)}dF_{T}^{0}(t)<\infty$ , support $\left(F_{X^{1}}\right)$ ⊆ support $\left(F_{X^{0}}\right)$ . Conditional mean embeddings^48^ are defined as kernel embeddings of conditional distributions:

(2) $$\begin{array}{rl} \mu_{T^{0}|X^{0}=x}(\cdot ) & :=E_{T^{0}|X^{0}}\left[l(T^{0},\cdot )|X^{0}=x\right] \end{array}$$

(3) $$\begin{array}{rl} & =\int_{0}^{\infty }l(t,\cdot )dF_{T^{0}|X^{0}=x}(t),\;x\in X \end{array}$$

See Muandet et al.^49^ for an extensive survey on the interpretation, estimation and properties of kernel—conditional and mean—embeddings. Another important kernel embedding is that of counterfactual distributions, known as Counterfactual Mean Embeddings^20^:

$$\mu_{T\langle 0|1\rangle }(\cdot )=\int_{0}^{\infty }l(t,\cdot )dF_{\left\langle 0|1\right\rangle }(t)$$

The previous definitions are reciprocally valid switching 0 by 1.

## 3. Empirical estimates of causal survival embeddings

We first discuss how to estimate $\mu_{T^{0}|X^{0}}=E_{T^{0}|X^{0}}[l(T^{0},\cdot )|X^{0}]:X⟶H$ . It is easy to see using the iterated expectations lemma and using conditional exogeneity that

$$\mu_{T\langle 0|1\rangle }(\cdot )=\int_{X}\mu_{T^{0}|X^{0}=x}(\cdot )dF_{X^{1}}(x)\in H$$

This suggests that upon obtaining ${\hat{\mu }}_{T^{0}|X^{0}=x}$ (simply done by isolating the data from control group), estimating Counterfactual Mean Embeddings is reduced to taking averages with respect to the covariates in the treatment group:

$${\hat{\mu }}_{T\langle 0|1\rangle }:=\frac{1}{m}\sum_{j=1}^{m}{\hat{\mu }}_{T^{0}|X^{0}=X_{j}^{1}}$$

In this section, we focus on $T^{0}$ and $X^{0}$ but, again, the same theory holds replacing 0 by 1 without loss of generality when it comes to estimate $\mu_{T\langle 1|0\rangle }$ . In Grünewälder et al.,^50^ the authors pose estimation of conditional mean embeddings in the absence of censoring as minimization of the theoretical risk $\tilde{R}(F)=E_{X^{0}}[\|\mu_{T^{0}|X^{0}}(X^{0})-F(X^{0}){\|}_{H}^{2}],\;F\in F$ where F is a vector-valued RKHS of functions X→H . For simplicity, we endow F with an H -kernel $\Gamma (x,x^{\prime })=k(x,x^{\prime })$ Id, where k is a scalar kernel on X and Id: H→H is the identity map on H . See the Supplemental material for a detailed explanation of vector-valued RKHSs and H -kernels.

We have by the generalized conditional Jensen’s inequality^51^ and iterated expectations lemma:

$$\begin{array}{rl} \tilde{R}(F) & =E_{X^{0}}[\|E_{T^{0}|X^{0}}[l(T^{0},\cdot )-F(X^{0})|X^{0}]{\|}_{H}^{2}]\\ & \leq E_{X^{0}}E_{T^{0}|X^{0}}[\|l(T^{0},\cdot )-F(X^{0}){\|}_{H}^{2}|X^{0}]\\ & =E_{T^{0}X^{0}}[\|l(T^{0},\cdot )-F(X^{0}){\|}_{H}^{2}]=:R(F) \end{array}$$

We will see that R(F) acts as a surrogate theoretical risk that admits an empirical version under right-censoring.

Now the problem is that, because of censoring, we do not have access to a sample from the joint distribution of $\left(T_{0},X_{0}\right)$ that would allow us to estimate the expectation involved by R(F) ; we instead observe times sampled from $\min\;\{T^{0},C^{0}\}$ . Let us further inspect the measure with respect to which the expectation in R(F) is taken:

$$\begin{array}{rl} dF_{T^{0}X^{0}}(t,x) & =P(T^{0}\in dt,X^{0}\in dx)\\ & =P(T\in dt,X\in dx|Z=0)\\ & =\frac{P(T\in dt,X\in dx|Z=0)P(Z=0)}{P(Z=0)}\\ & =\frac{P(\Delta =1,T\in dt,X\in dx,Z=0)}{P(Z=0)P(\Delta =1|T=t,X=x,Z=0)}\\ & =\frac{P(\Delta =1,T\in dt,X\in dx|Z=0)}{P(\Delta =1|T=t,X=x,Z=0)}\\ & =\frac{dF_{0}^{\left(*\right)}(t,x)}{G_{0}(t,x)} \end{array}$$

where $G_{0}(t,x)=P(\Delta =1|T=t,X=x,Z=0)$ is the conditional probability that an observation is uncensored given that the event time is t and the covariates are x conditional on the control population and $F_{0}^{\left(*\right)}(t,x)=P(\Delta =1,T\leq t,X\leq x|Z\;=\;0)$ is the law of uncensored observations in the control population.^52,53^

Note that if we assume C⊥⊥T|Z and Δ⊥⊥X|T,Z then $G_{0}(t,x)=P(\Delta =1|T=t,X=x,Z\;=\;0)=P(\Delta =1|T\;=\;t,Z\;=\;0)=P(C>t|Z=0)$ and therefore $G_{0}(t,x)=G_{0}(t)$ equals 1− the marginal law of censoring times conditional on Z=0 .

Let $\left(T_{1}^{*},\Delta_{1},0,X_{1}),\ldots ,(T_{n}^{*},\Delta_{n},0,X_{n}\right)$ be i.i.d. observations from the control group Z=0 . By plugging in an estimate ${\hat{G}}_{0}(t,x)$ and the empirical measure

$$d{\hat{F}}_{0}^{\left(*\right)}(t,x)=\frac{1}{n}\sum_{i=1}^{n}\Delta_{i}\delta_{T_{i}^{*}}(t)\delta_{X_{i}}(x)$$

we arrive to a regularized empirical risk minimization problem:

$$\begin{array}{rl} {\hat{R}}_{\varepsilon ,n}(F) & :=\frac{1}{n}\sum_{i=1}^{n}\frac{\Delta_{i}}{{\hat{G}}_{0}(T_{i}^{*},X_{i})}{\left\|l\left(T_{i}^{*},\cdot \right)-F\left(X_{i}\right)\right\|}_{H}^{2}\\ & \;+\varepsilon \|F{\|}_{F}^{2} \end{array}$$

We denote its minimizer by μ^ε,n:=argminF∈FsR^ε,n(F) . We define $W_{i}:=\frac{\Delta_{i}}{{\hat{G}}_{0}(T_{i}^{*},X_{i})}$ In the following, we derive a closed expression for ${\hat{\mu }}_{\varepsilon ,n}$ .

Lemma 1 A minimizer of the empirical risk ${\hat{R}}_{\varepsilon ,n}(F)$ is unique and can be expressed as $\sum_{j=1}^{n}\Gamma (\cdot ,X_{i})(c_{i})$ where the coefficients $\{c_{j}:j=1,\ldots ,n\}\subseteq H$ are the unique solution of the linear equations $\sum_{j=1}^{n}(W_{i}\Gamma (X_{i},X_{j})+n\varepsilon \delta_{ij})(c_{j})=W_{i}h_{i},i=1,\ldots ,n$ .

Proof. See Supplemental material.  □

Choosing $\Gamma (x,x^{\prime })=k(x,x^{\prime })\;\mathrm{Id}$ (see Grünewälder et al.^50^ for more details on why this is a sensible election) we conclude that the coefficients $(c_{1}\ldots c_{n}{)}^{\prime }=:C$ showing up in Lemma 1 can be computed as $WH=(WK+n\varepsilon I)C⟺C=(WK+n\varepsilon I{)}^{-1}WH$ , where $K_{ij}=k(X_{i},X_{j})$ , $W=\text{diag}(W_{1},\ldots ,W_{n})$ , $H=(h_{1}\ldots h_{n}{)}^{\prime }$ , $C=(c_{1}\ldots c_{n}{)}^{\prime }$ . Now the *conditional* mean embedding evaluated on the covariates of the treated sample $\left(X_{1}^{1},\ldots ,X_{m}^{1}\right)$ is $(\hat{F}(X_{1}^{1})\ldots \hat{F}(X_{m}^{1}))=(\sum_{j=1}^{n}k(X_{1}^{1},X_{j})c_{j}\ldots \sum_{j=1}^{n}k(X_{m}^{1},X_{j})c_{j})=C^{\prime }\tilde{K}$ where ${\tilde{K}}_{ij}=k(X_{i},X_{j}^{1})$ .

The *counterfactual* mean embedding is computed by taking the average of the previous row: ${\hat{\mu }}_{T\langle 0|1\rangle }(\cdot )=C^{\prime }\tilde{K}1_{m}$ where $1_{m}$ is a vector of all ones divided by m . By recovering the expression of C previously derived we have a closed expression for the *counterfactual* mean embedding estimator

$${\hat{\mu }}_{T\langle 0|1\rangle }(\cdot )=H^{\prime }W(KW+n\varepsilon I{)}^{-1}\tilde{K}1_{m}$$

and its row-shaped version (visually, resembles better to a function of time) is

$${\hat{\mu }}_{T\langle 0|1\rangle }^{\prime }(\cdot )=1_{m}^{\prime }{\tilde{K}}^{\prime }(WK+n\varepsilon I{)}^{-1}WH$$

It is important to bear in mind that $H=(l(T_{1}^{*},\cdot ),\ldots ,l(T_{n}^{*},\cdot ){)}^{\prime }$ . We can always evaluate $H_{ij}=l(T_{i}^{*},t_{j})$ on a grid $t_{1},\ldots ,t_{N}$ .

Remark Computing an estimator of $P[{\tilde{T}}^{1}>t]-P[{\tilde{T}}^{0}>t]$ is possible once ${\hat{S}}_{T\langle 0|1\rangle }$ and ${\hat{S}}_{T\langle 1|0\rangle }$ have been reconstructed following Section 4 in the Supplemental material from ${\hat{\mu }}_{T\langle 0|1\rangle }$ and ${\hat{\mu }}_{T\langle 1|0\rangle }$ . By the law of total probability,

$$\begin{array}{rl} P\left[{\tilde{T}}^{1}>t\right]-P\left[{\tilde{T}}^{0}>t\right] & =\left(P\left[{\tilde{T}}^{1}>t|Z=0\right]-P\left[{\tilde{T}}^{0}>t|Z=0\right]\right)P(Z=0)\\ & \;+\left(P\left[{\tilde{T}}^{1}>t|Z=1\right]-P\left[{\tilde{T}}^{0}>t|Z=1\right]\right)P(Z=1) \end{array}$$

By virtue of Chernozhukov et al.^16^ and assuming conditional exogeneity, we have:

$$\begin{array}{rl} P\left[{\tilde{T}}^{1}>t\right]-P\left[{\tilde{T}}^{0}>t\right] & =(S_{T\langle 1|0\rangle }-S_{T\langle 0|0\rangle })P(Z=0)\\ & \;+(S_{T\langle 1|1\rangle }-S_{T\langle 0|1\rangle })P(Z=1) \end{array}$$

This formulation enables the estimation of the counterfactual survival probability gain across arms using CSEs, our approach, by replacing the terms in the expression above with their empirical counterparts.

## 4. Asymptotics of causal survival embeddings

This section comprises the main theoretical contribution of our work. Let us get started by a couple of definitions needed to reexpress parameters and their estimators in a more convenient way regarding proofs.

### 4.1. Population and empirical covariance operators

We need to introduce the following definitions^20,54^ in order to state theoretical results regarding asymptotic properties of our estimator.

Definition 2 Let $C_{TX}:G\to H$ be the covariance operator of the random variables $X^{0}$ and $T^{0}$ defined as

$$C_{TX}f=E_{X^{0}T^{0}}\left[l\left(\cdot ,T^{0}\right)f\left(X^{0}\right)\right],\;f\in G$$

Substituting in the measure $dF_{X^{0}T^{0}}=\frac{dF_{0}^{\left(*\right)}}{G_{0}}$ the empirical counterparts ${\hat{F}}_{0}^{\left(*\right)}$ and ${\hat{G}}_{0}$ we obtain

Definition 3 Let $\left(X_{1},T_{1}^{*}),\ldots ,(X_{n},T_{n}^{*}\right)$ be i.i.d. observations from the control group Z=0 . We define the censored empirical covariance operator as:

$$\begin{array}{rl} {\hat{C}}_{XX}^{*}f & :=\frac{1}{n}\sum_{i=1}^{n}W_{i}k\left(\cdot ,X_{i}\right)f\left(X_{i}\right)\\ {\hat{C}}_{TX}^{*}f & =\frac{1}{n}\sum_{i=1}^{n}W_{i}l\left(\cdot ,T_{i}^{*}\right)f\left(X_{i}\right),\;f\in G \end{array}$$

The following result shows that we can write ${\hat{\mu }}_{T\langle 0|1\rangle }$ using the empirical covariance operators.

Lemma 2 Let ${\hat{\mu }}_{X_{1}}$ the kernel mean embedding estimated with the sample covariates from the treated population. Then we have

$${\hat{\mu }}_{T\langle 0|1\rangle }={\hat{C}}_{TX}^{*}{\left({\hat{C}}_{XX}^{*}+\varepsilon I\right)}^{-1}{\hat{\mu }}_{X_{1}}$$

Proof. See Supplemental material.  □

### 4.2. Assumptions

In the following, we introduce the assumptions needed for establishing consistency of our proposed estimator. Recall that l and k are kernels on (0,+∞) and covariate space, respectively.

1.
$\underset{x\in X}{\sup}k(x,x)<\infty$ and $\underset{t\in (0,+\infty )}{\sup}l(t,t)<\infty$
2.
$\text{The RKHS }H\text{ of }k\text{ is dense in }L_{2}(F_{X^{0}})\text{. }$ This is particularly satisfied by Gaussian kernels.^55^
3. The distribution $F_{X^{1}}$ is absolutely continuous with respect to $F_{X^{0}}$ with the Radon-Nikodym derivative $g:=dF_{X^{1}}/dF_{X^{0}}$ satisfying $g\in L_{2}(F_{X^{0}})$ . It also implies the support equality condition used throughout the paper.
4.
$\left(T_{1}^{*},\Delta_{1},0,X_{1}),\ldots ,(T_{n}^{*},\Delta_{n},0,X_{n}\right)$ are i.i.d. observations from the control group, and $X_{1}^{1}\ldots ,X_{m}^{1}$ are i.i.d. observations of the random variable $X^{1}$ .
5.
C⊥⊥T|Z (independence) and Δ⊥⊥X|T,Z (conditional independence of the censoring indicator and the covariates given the realized time). This automatically implies
$$\begin{array}{rl} G_{0}(t,x) & =P(C>t|T=t,X=x,Z=z)\\ & =P(C>t|T=t)=P(C>t)=:G_{0}(t) \end{array}$$
  In this case, it is possible to estimate $G_{0}(t)$ using the marginal reverse Kaplan–Meier estimator- flipping the event indicators and using the canonical Kaplan–Meier estimator.^56^ See Stute^57^ and Stute^52^ for further comments.
6.
$$\frac{1}{G_{0}^{2}}\text{ and a.s. }\frac{1}{{\hat{G}}_{0}^{2}}<\infty$$
  This ensures that population and empirical covariance operators are well defined as Bochner integrals.^58^

### 4.3. Consistency and convergence rate

Our main theoretical contribution is the convergence rate of the stochastic error in RKHS norm in Theorem 1. Once established, we use it to establish consistency in Corollary 1 and find the final convergence rate in Corollary 2.

Theorem 1 **Convergence rate of the stochastic error**

Consider the CSEs estimator ${\hat{\mu }}_{T\langle 0|1\rangle }$ . Rename the regularization constant $\varepsilon \equiv \varepsilon_{n}>0$ . Suppose that Assumptions (i.) to (vi.) hold ((ii.) is optional). Then we have for the stochastic error

$$\begin{array}{rl} & {\left\|{\hat{C^{*}}}_{TX}({\hat{C^{*}}}_{XX}+\varepsilon_{n}I{)}^{-1}{\hat{\mu }}_{X_{1}}-C_{TX}(C_{XX}+\varepsilon_{n}I{)}^{-1}\mu_{X_{1}}\right\|}_{H}\\ & \;=O_{P}\left(n^{-1/2}\varepsilon_{n}^{-1}\right) \end{array}$$

Proof. See Supplemental material.  □

Corollary 1 **Consistency**

Suppose that Assumptions (i.) to (vi.) are satisfied. Let the regularization constant $\varepsilon \equiv \varepsilon_{n}>0$ depend on sample size. Then if $\varepsilon_{n}\to 0$ and $n^{1/2}\varepsilon_{n}\to \infty$ as n→∞ , we have

$${\left\|{\hat{\mu }}_{T\langle 0|1\rangle }-\mu_{T\langle 0|1\rangle }\right\|}_{H}\to 0$$

in probability as n→∞ .

Proof. See Supplemental material.  □

Corollary 2 **Convergence rate**

Suppose that Assumptions (i.) to (vi.) in our paper and Assumptions 3 and 4 in Muandet et al.^20^ hold with α+β≤1 both non-negative. Let $\varepsilon_{n}>0$ be a regularization constant. Let c>0 be an arbitrary constant, and set $\varepsilon_{n}=cn^{-1/(1+\beta +\max(1-\alpha ,\alpha ))}$ . Then we have

$${\left\|{\hat{\mu }}_{T\langle 0|1\rangle }-\mu_{T\langle 0|1\rangle }\right\|}_{H}=O_{p}\left(n^{-(\alpha +\beta )/2(1+\beta +\max(1-\alpha ,\alpha ))}\right)$$

Proof. See Supplemental material.  □

Informally, α and β quantify respectively how similar are $F_{X^{0}}$ and $F_{X^{1}}$ (the bigger α , the more similar) and the smoothness of the map $x\mapsto \mu_{T^{0}|X^{0}=x}$ (the bigger β , the smoother).

## 5. Numerical experiments

In this section, we thoroughly evaluate the effectiveness of CSEs through three focused analyses. First, we conduct an empirical comparison with state-of-the-art methods to benchmark CSEs against existing techniques (Section 5.1). Second, we assess the asymptotics of our estimator itself at the RKHS scale, examining its performance across various sample sizes and simulation runs (Section 5.2). Third, we evaluate the reconstruction of counterfactual survival functions through CSEs at the raw distributional scale under different levels of censoring and distributional shifts (Section 5.3). This structured approach allows us to demonstrate the advantages of the new method compared to existing approaches and subsequently verify its convergence properties.

### 5.1. Empirical comparison with the literature

We present a comparative analysis of four methods—CSEs, Counterfactual Mean Embeddings^20^ considering uncensored observations, AKME, and Causal Survival Forests (GRF)—in estimating survival functions. We simulate 1000 times a realistic survival analysis scenario with confounding due to the shifts in covariate distributions and complex, non-linear relationships between covariates and survival times (see 5.1.4).

#### 5.1.1. Causal survival forests

Recall the parameter of interest, $P({\tilde{T}}^{1}>t)-P({\tilde{T}}^{0}>t)=:d(t)$ for t≥0 . Causal Survival Forests^33^ aim to estimate the effect of Z on a deterministic transformation y of the survival time:

$$\tau (x)=E\left[y\left({\tilde{T}}^{1}\right)-y\left({\tilde{T}}^{0}\right)|X=x\right]$$

By choosing $y_{t}(T)=1\{T>t\}$ and using linearity of expectations, the estimand above becomes:

$$\tau_{t}(x)=P\left[{\tilde{T}}^{1}>t|X=x\right]-P\left[{\tilde{T}}^{0}>t|X=x\right]$$

so that our initial parameter of interest can be estimated using Causal Survival Forests noting that $d(t)=E[\tau_{t}(X)]$ .

Remark Choosing a different value of t>0 changes the estimand for Causal Survival Forests, requiring a separate run for each specific time point. Consequently, this approach becomes significantly more computationally demanding, as multiple estimations are necessary to cover different time horizons. For example, using a grid of 50 time horizons, GRF took 31.831 seconds (elapsed time), whereas Causal Survival Embeddings required only 0.621 seconds.

#### 5.1.2. Inverse probability of treatment weighting based Kaplan–Meier estimator

Inverse probability of treatment weighting is among the various approaches that leverage the treatment assignment mechanism to adjust for confounding. Specifically, Xie and Liu^32^ proposed an IPTW-based Kaplan–Meier estimator, referred to as the AKME estimator, which applies weights derived from the inverse probability of group assignment to adjust survival estimates.

#### 5.1.3. Counterfactual mean embeddings (Naive)

The Naive method we define involves fitting Counterfactual Mean Embeddings^20^ using only non-censored observations. This approach is commonly adopted when attempting to apply methods originally designed for complete (non-censored) data to survival analysis settings.^59^ Have into account that the Naive method does not borrow information from censored observations and results therefore in an inefficient estimator.

#### 5.1.4. Simulation setup

We generated synthetic data to compare the performance of the described methods with our approach. A single dataset consists of two groups: A control group with n=1000 observations and a treatment group with m=800 observations.

Each observation includes three covariates $X_{1}$ , $X_{2}$ , and $X_{3}$ . For the control group, the covariates are independently drawn from a normal distribution with mean 1 and standard deviation σ=1 :

$$X_{j,\text{control}}∼N(1,1),\;j=1,2,3$$

For the treatment group, we introduced shifts to simulate confounding effects:

$$X_{j,\text{treatment}}∼N(1+\delta_{j},1),\;j=1,2,3$$

where the shifts are $\delta_{1}=1$ , $\delta_{2}=-0.5$ , and $\delta_{3}=0.1$ . This creates differing covariate distributions between the control and treatment groups, introducing confounding into the simulation.

The population conditional survival functions for the control and treatment groups are non-linear functions of the covariates, incorporating complex interactions to reflect realistic scenarios. For the control group:

$$T_{\text{control}}∼\text{Exponential}\left(\lambda_{\text{control}}\right),\;\lambda_{\text{control}}=\frac{1}{r_{\text{control}}+1}$$

where

$$r_{\text{control}}=\exp\;\left(-{\left[\beta_{0,1}\left(\pi X_{1}X_{2}\right)+\beta_{0,1}X_{3}^{3}\right]}^{2}\right)$$

and $\beta_{0}=(-0.2,0.3)$ . This function introduces interaction between $X_{1}$ and $X_{2}$ and a non-linear effect of $X_{3}$ .

For the treatment group, we apply a time shift factor s=2 to model the treatment effect:

$$T_{\text{treatment}}∼\text{Exponential}\left(\lambda_{\text{treatment}}\right),\;\lambda_{\text{treatment}}=\frac{1}{s(r_{\text{treatment}}+1)}$$

where

$$r_{\text{treatment}}=\exp\;\left(-{\left[\beta_{1,1}\cos\;\left(\pi X_{2}X_{3}\right)+\beta_{1,2}X_{1}^{2}\right]}^{2}\right)$$

with $\beta_{1}=(-0.3,0.1)$ . This function includes a cosine interaction between $X_{2}$ and $X_{3}$ and a squared term of $X_{1}$ , adding further non-linearity.

For both groups, censoring times follow exponential distributions:

$$\begin{array}{rl} C_{\text{control}} & ∼\text{Exponential}\left(\delta_{0}\right),\;\delta_{0}=1.75\\ C_{\text{treatment}} & ∼\text{Exponential}\left(\delta_{1}\right),\;\delta_{1}=1.5 \end{array}$$

We use linear logistic regression to estimate the propensity scores required for weighting in the AKME. This approach is appropriate because $X^{0}$ and $X^{1}$ follow shifted Gaussian distributions with respect to each other, which allows for an effective separation using a linear decision boundary. The AKME is implemented using the R package adjustedCurves.^60^ For the Causal Survival Forests, we employed the grf package, specifically utilizing the function causal_survival_forest. The Naive (see 5.1.3) approach is easily implemented by setting all the weights $W_{i}=\Delta_{i}$ in our implementation of CSEs.

All estimates are assessed by comparison with the true counterfactual survival difference $d(t)=P({\tilde{T}}^{1}>t)-P({\tilde{T}}^{0}>t)$ . Our primary focus is on evaluating the estimators’ performance across the entire survival curves, as in Denz et al.^60^

Following Klein and Moeschberger,^61^ we consider the mean-squared error (MSE) between the estimated and true survival curves. For a given simulation run,

$${\hat{\Delta }}_{\mathrm{MSE}}=\int_{0}^{\tau }{\left(d(t)-\hat{d}(t)\right)}^{2}dt$$

where τ is the 95% quantile of the true survival times.

**Figure 1.**
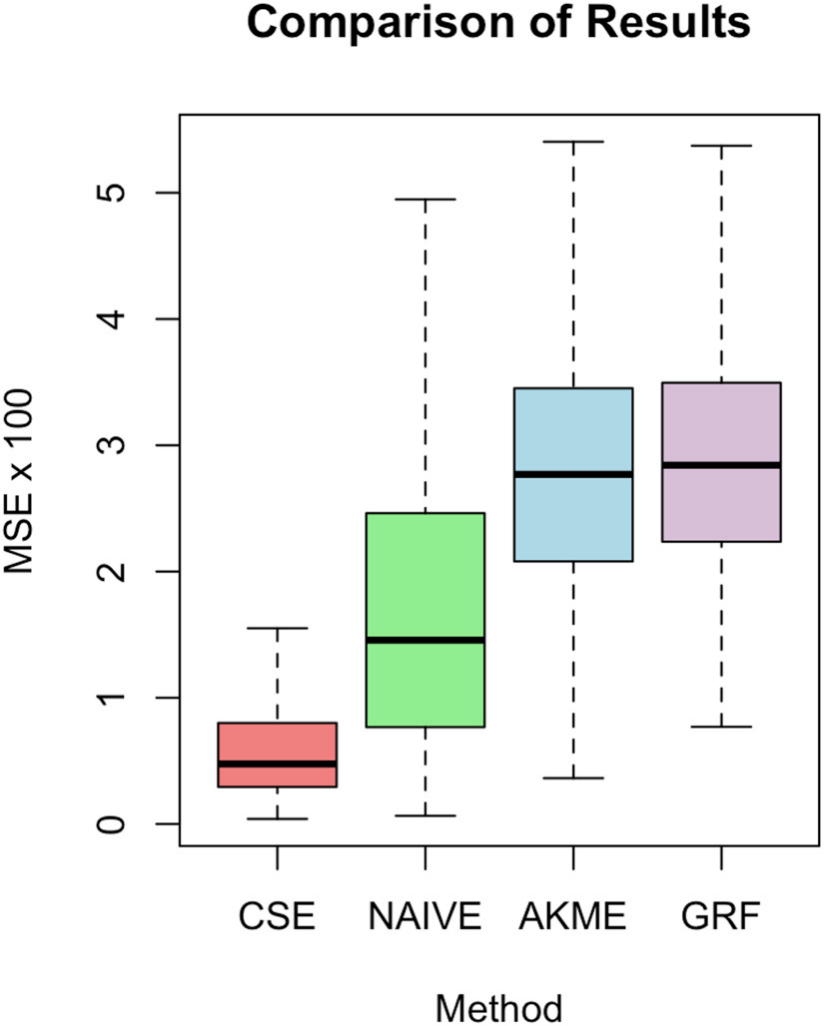
Comparison of our approach with the three methods described in Section 5.1.

**Figure 2.**
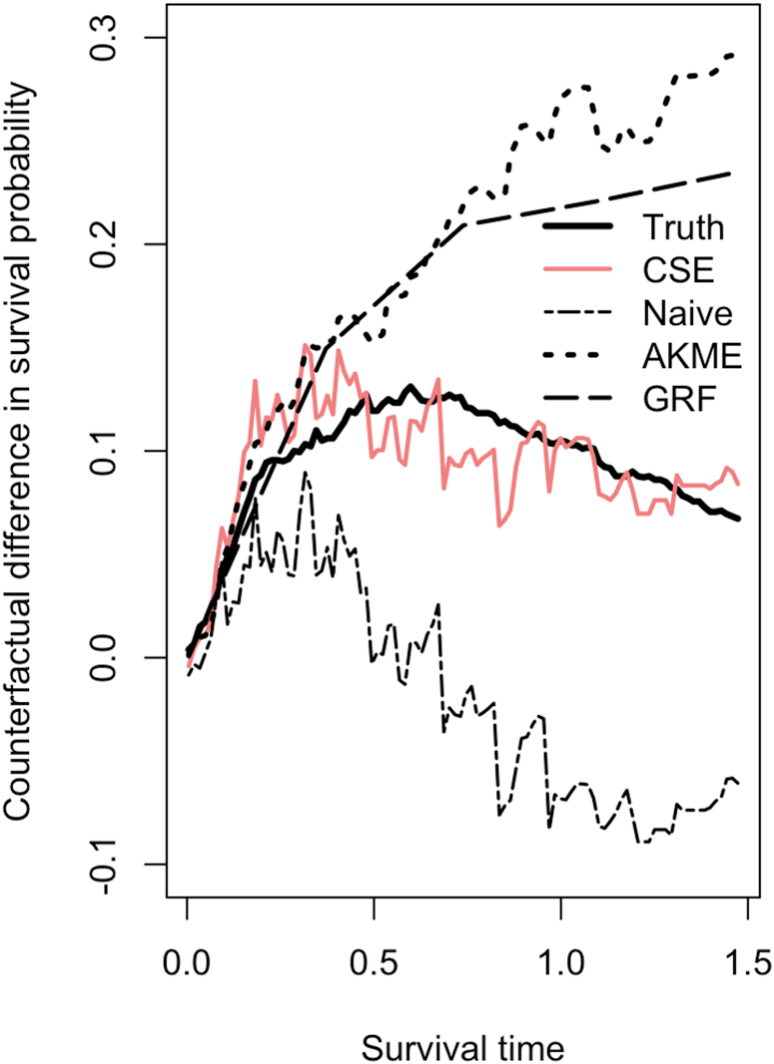
The true difference between counterfactual survival functions, d(t) , is represented by the solid black line.

We compute the arithmetic mean and standard deviation of ${\hat{\Delta }}_{\mathrm{MSE}}$ , both multiplied by 100 for readability, over 1000 simulation repetitions and display the results in Table 1 and Figure 1. The results from the comparative experiments in this section are visible in Figures 1 and 2 and Table 1.

**Table 1.** Comparison of results: MSE average and standard deviation.

Method	Average ( ×100 )	Std Dev. ( ×100 )
Ours	0.621	0.521
Naive	1.734	1.264
AKME	2.825	1.013
Causal Surv. Forest	2.919	0.957

Observe that Causal Survival Embeddings perform almost five times better in terms of average and twice better in terms of standard deviation better than Causal Survival Forests. It is natural that the Naive estimator shows less stability as it only considers uncensored observations. Percentage of observed events conditional on Z=0 : approximately 30%. Percentage of observed events conditional on Z=1 : approximately 15%. AKME: Adjusted Kaplan–Meier Estimator; MSE: mean-squared error.

The superior performance of CSE can be attributed to several factors. While both CSE and GRF utilize flexible, non-parametric approaches to model the survival function and capture complex relationships between covariates and survival times, there are some notable distinctions in their underlying assumptions and analytical frameworks.

CSE directly employs conditional mean embeddings without requiring specific functional assumptions about the propensity score. GRF, built on the random forest framework, also models complex relationships through an ensemble of decision trees that partition the feature space. While random forests are technically non-parametric, their theoretical analysis often involves simplifying assumptions. Specifically, for studying the asymptotic properties of random forests, an additive regression model for the response is frequently assumed.^62^ This assumption helps to establish theoretical guarantees regarding asymptotics but can limit the flexibility of the model when applied to data with non-additive or non-linear interactions between covariates and outcomes. As detailed in Scornet et al.,^62^ this additive assumption aids in understanding the behavior of random forests in large samples but may not always align with the model’s practical application, especially when the true relationships are more complex.

Secondly, unlike AKME, which uses IPTW to adjust for treatment assignment, CSE does not require reweighting with propensity-to-treatment scores. Reweighting can introduce additional variance, especially in regions with few data or extreme propensity scores.

Thirdly, CSE inherently accounts for censoring without excluding censored observations. This contrasts with the Naive method, which only considers uncensored data, leading to higher variability because of small sample sizes, particularly at larger time horizons. The ability to incorporate censored data allows CSE to maintain accuracy across the entire range of observed times.

In conclusion, our proposal demonstrates clear advantage over the other methods evaluated in this setting.

### 5.2. Embeddings estimator consistency

**Figure 3.**
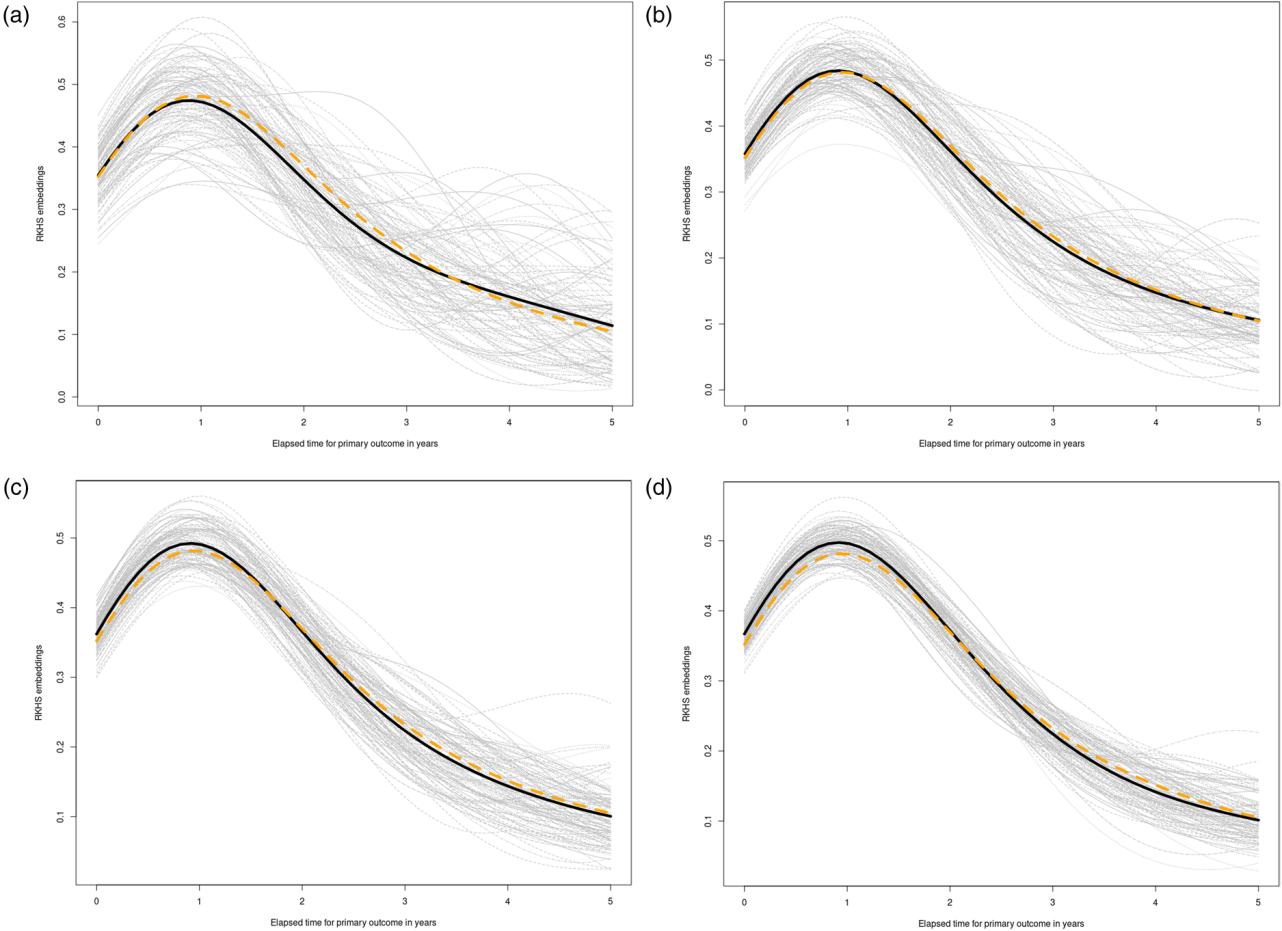
Simulation results for subsection 5.2. See setup details in the Supplemental material (subsection 7.2). (a) n=100 ; (b) n=200 ; (c) n=300 ; (d) n=500 .

We perform estimation of CSEs for 100 different simulation runs in four different sample sizes scenarios. The results are displayed in Figure 3. The black solid line represents the average of the 100 CSEs ${\hat{\mu }}_{T\langle 0|1\rangle }$ estimators (Section 5.2). The dashed yellow line is a numerical approximation of the population counterfactual mean embedding. Due to the analytical complexity of the integrals involved in the population definition of CSEs (see Supplemental material), we have opted to proceed with a Monte Carlo approximation for avoiding the limitations imposed by an explicit formulation in this context. Each gray line corresponds with one simulation draw. Simulation parameters were tuned by hand in order to set a censoring percentage of approximately 75% (on average across simulations, only 25% of information was complete). m has the same value of n in all cases.

### 5.3. Survival functions estimator consistency

We undertake an examination of the impact of varying levels of censoring percentage on the reconstruction of counterfactual survival functions. To achieve this, we maintain a fixed sample size— 800 observations in the control arm and 800 observations in the treatment arm—while systematically tuning the proportion of complete information available. This setup aims to shed light on how different degrees of censoring influence the accuracy and reliability of our method for estimating counterfactual survival functions.

Our simulation design encompasses two distinct scenarios, each representing a fundamental aspect of observational studies. In the first scenario, we delve into cases where observational differences across treatment groups are primarily driven by distributional variations within the treated arm, corresponding (Case I in the Supplemental material). In the second scenario, we consider situations where disparities in survival outcomes between treatment arms arise predominantly from shifts in the covariate distribution (Case II in Supplemental material).

**Figure 4.**
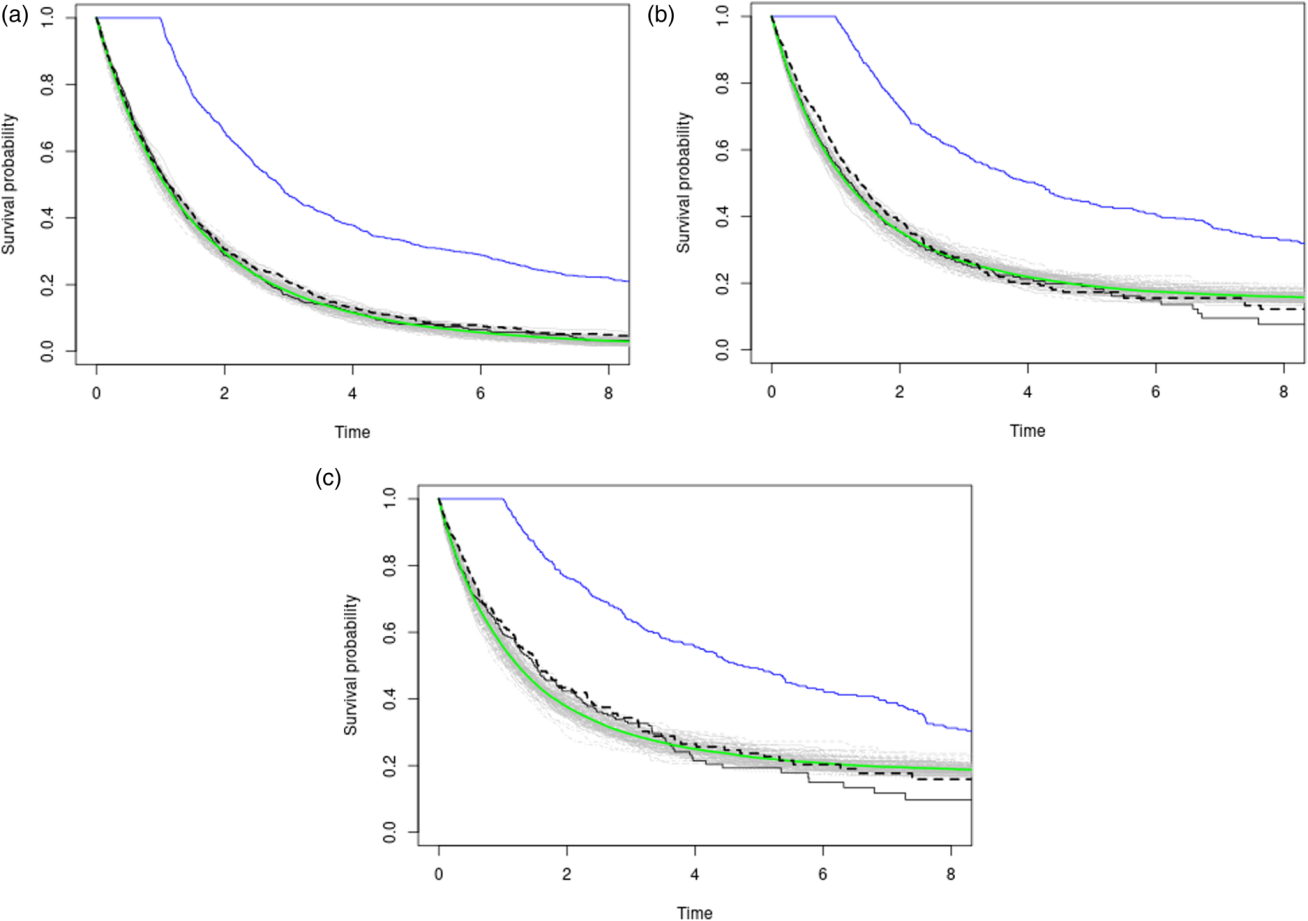
Three plots with varying censoring percentages (approx. 25%, 50%, 65% in descending order) in a no-confounding scenario on a fixed grid of time-points. The black and blue curves represent observational Kaplan–Meier estimates for the placebo and treatment arms, respectively. The gray lines depict 100 Causal Survival Embeddings obtained from simulation runs, while the green line represents the average. The dashed line represents the empirical distribution function of placebo counterfactual times in Z=1 . Following the decomposition in equation (1), the dashed (only accessible through simulation) and green (aiming to estimate the dashed) lines should closely resemble the solid black line. We see that our estimator shows a correct behavior because the gray simulated bands tend to capture the dashed line.

Results are displayed in Table 2 and a graphical depiction of the simulation results is in Figure 4. Further details on the simulation process can be found in Supplemental material 7.3.

**Table 2.** Performance metrics for different censoring percentages.

Censoring Percentage (approx.)	65%	50%	25%
Case I	$2.53\times 10^{-2}$	$1.65\times 10^{-2}$	$1.93\times 10^{-5}$
Case II	$2.13\times 10^{-2}$	$1.09\times 10^{-2}$	$4.36\times 10^{-3}$

Notice how accuracy diminishes with censoring as expected.

## 6. Application to SPRINT: A landmark trial in public health

NIH’s Systolic Blood Pressure Intervention Trial (SPRINT) was conducted to inform the new blood pressure medication guidelines in the US by testing the effects that a lower blood pressure target has on reducing heart disease risk. Observational studies had shown that individuals with lower systolic blood pressure (SBP) levels had fewer complications and deaths due to cardiovascular disease (CVD). Building on this observation, the NIH’s Systolic Blood Pressure Intervention Trial (SPRINT) was designed to test the effects of a lower blood pressure target on reducing heart disease risk. Specifically, SPRINT aimed to compare treating high blood pressure to a target SBP goal of less than 120 mmHg against treating to a goal of less than 140 mmHg.

However, it has been seen in major clinical trials that a reduction of SBP is intimately connected to a reduction of diastolic blood pressure (DBP). Despite this association, it is debated whether low DBP leads to undesirable cardiovascular outcomes, such as a reduction of coronary flow, myocardial infarction, heart failure, or cardiovascular death.^63–65^ This suggests that intensive SBP therapy may result in an excessive reduction of DBP and therefore result in an undesired increase in cardiovascular risk. Nevertheless, SPRINT showed that intensive treatment was clearly associated with a reduced risk of CVD and was even finished early because its results were so convincing.^31^ Given the conclusions drawn by SPRINT, the research question is now whether it is possible to decompose the total effect of treatment on the primary outcome into a (natural) direct effect and a (natural) indirect effect through low DBP (induced by the treatment).

The debate on intensive blood pressure therapy is ongoing. Lee et al.^66^ set out to ascertain whether there is an association between the onset of diastolic hypotension during treatment and negative outcomes. To achieve this, they utilized a conventional Cox PH model, using DBP as a time-varying exposure and adjusting for certain baseline factors. Stensrud et al.^67^ aimed to explore whether a formal mediation analysis, utilizing the SPRINT data, could identify whether intensive SBP treatment impacts cardiovascular outcomes via a pathway that involves DBP below 60 mmHg. They claim that *the association between treatment-induced DBP and cardiovascular outcomes suffers from confounding*.^68^

We illustrate how our methodological contribution manages to perform the desired effect decomposition both across pathways and, importantly, across time thanks to the RKHS formulation. A consensus answer to the problem would be relevant to the medical community because, as mentioned, SPRINT ultimately informed the new blood pressure guidelines by demonstrating that a lower blood pressure target can significantly reduce heart disease risk.

### 6.1. Naive analysis of SPRINT

We might start by stratifying the observations into two groups: One with DBP ≤60 mmHg one year after randomization (encoded *DBP60=0*) and a group with >60 mmHg one year after randomization (encoded *DBP60=1*). Then we regress the primary endpoint against the newly created indicator variable using vanilla Cox PH (see details in the Supplemental material).

The estimates provided by the model fit would confirm the original suspicions of the medical community, stating that low DBP leads to increased cardiovascular risk. This is because the estimate of the hazard ratio *exp(coef)=1.326* >1 .

**Figure 5.**
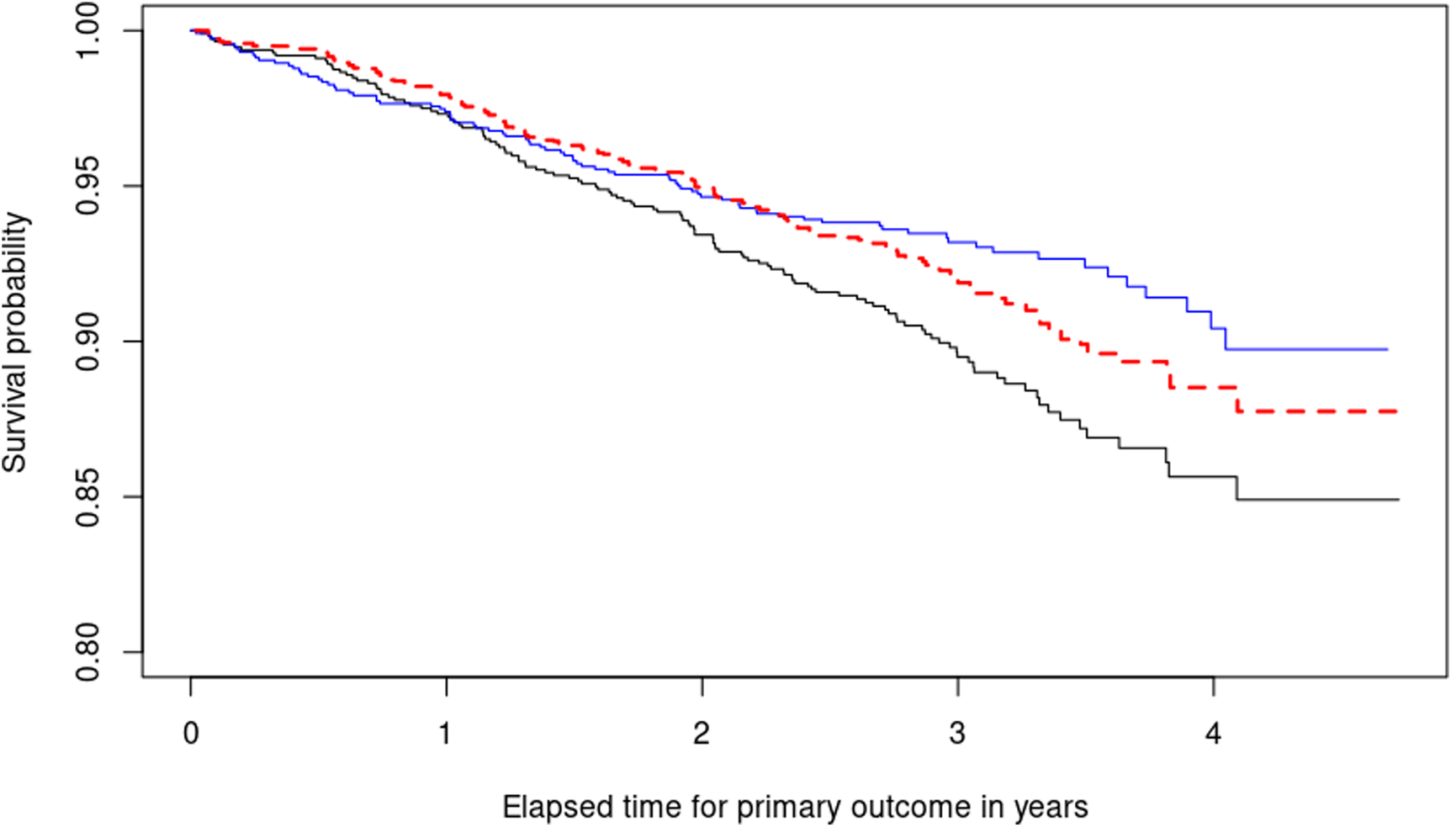
Illustrating the decomposition of treatment effects using equation (1). Two Kaplan–Meier survival curves are shown: The blue curve represents the estimated survival probability $S_{{\tilde{T}}^{1}|Z=1}(t)$ based on observational data from the treatment group, and the black curve represents $S_{{\tilde{T}}^{0}|Z=0}(t)$ from the control group. In equation (1), (B) is zero if and only if the outcome disparity is due to distributional differences between the covariates across arms. The dashed red line represents our proposed estimator for $S_{T\langle 0|1\rangle }$ , the remaining component in equation (1).

The second step we take is to fit two Kaplan–Meier curves, one for each arm of the SPRINT trial (*INTENSIVE=0* target SBP of 140 mmHg, *INTENSIVE=1* target SBP of 120 mmHg) and produce the black and blue lines respectively displayed in Figure 5. This serves as a quantitative basis for three facts. First, the paradox we are facing becomes empirically confirmed because now treatment defined as SBP lowering intervention seems to be effective (the blue curve therein estimating the survival function of the treatment population is higher after one year). Second, the estimates of the survival functions are crossing. This is a well-known problem in the field of time-to-event analysis,^69^ directly invalidating the proportional hazards assumption. Third, this would confirm observationally the overall positive results of the SPRINT trial, asserting that intensive SBP control results in cardiovascular benefit.

### 6.2. Conclusions of our analysis of SPRINT

Our response variable *T_PRIMARY* is observed time-to-primary outcome in days. The treatment indicator for each patient *INTENSIVE* is encoded such that 1 indicates lower SBP target of 120 mmHg and 0 indicates standard treatment (target SBP: 140 mmHg). The vector of covariates for each patient includes *‘‘DBP.1yr’’* (DBP one year after randomization) and baseline characteristics we want to adjust for: *‘‘DBP.rz’’* DBP at randomization and *‘‘AGE’’*

Our results agree with Stensrud and Strohmaier^67^ and Beddhu et al.^70^: The increased risk in subjects with diastolic pressure below 60 cannot be fully explained by the intensive treatment itself, but may be due to other factors. A complete description of the results is included in Figure 5. For most of the study duration (approximately from year 1 onwards), intensive reduction of SBP counteracts the harmful consequences of reduced DBP, leading to increased survival in the treatment arm, as evidenced by the blue curve being above the black one. However, the difference between $S_{T^{1}}(t)$ and $S_{T^{0}}(t)$ is primarily driven by DBP reduction for a period around one to two years, where $S_{T\langle 0|1\rangle }\approx S_{T\langle 1|1\rangle }$ . Interestingly, the increased survival probability in the SBP arm starting from year 2 is due to reductions in both SBP (encoded in the treatment indicator) and DBP (encoded in the covariates). This happens because for t greater than $t_{0}\in (3,3.5)$ years, $S_{T\langle 0|1\rangle }-S_{T\langle 0|0\rangle }(t)\approx S_{T\langle 1|1\rangle }-S_{T\langle 0|1\rangle }(t)$ . In summary, the treatment effect is not solely attributed to intensive SBP reduction, and the plot in Figure 5 breaks down the origins of observational differences over time.

Specifically, our novel estimator provides valuable insights that support the findings in Stensrud and Strohmaier,^67^ which suggest that the association between treatment-induced DBP and cardiovascular outcomes may be confounded. This corroborates similar findings reported in the literature, as mentioned in the study,^70^ which align with our own results.

When comparing our proposed method with existing non-parametric estimators in the SPRINT trial application case, we benchmark CSEs against Causal Survival Forests.^33^ We selected a fully non-parametric estimator to avoid the need for specifying a propensity score model.

**Figure 6.**
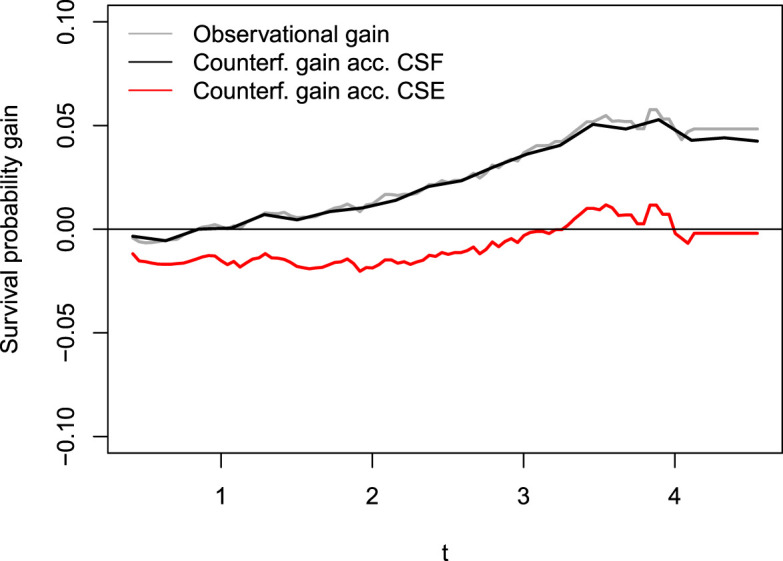
Difference between conditional KM estimators (gray) and estimations of counterfactual survival probability gain, $P({\tilde{T}}^{1}>t)-P({\tilde{T}}^{0}>t)$ , for t>0 . Causal Survival Forests align with the observational, confounded difference and cannot isolate the effect of DBP. Results indicate that SBP intervention, when detached from the DBP reduction that comes by hand, reduces cardiac complication risk only in the long term. KM: Kaplan–Meier; DBP: diastolic blood pressure; SBP: systolic blood pressure.

In Figure 6, we display in gray the differences between the arm-conditioned Kaplan–Meier (KM) estimators (blue minus black lines in Figure 5) alongside an estimate $\hat{d}(t)$ of the counterfactual survival probability gain, $P({\tilde{T}}^{1}>t)-P({\tilde{T}}^{0}>t),t>0$ . Our results corroborate existing literature: Counterfactually comparing a scenario where *everyone* has their SBP lowered ( Z=1 ) with a scenario where *no one* has their SBP altered shows that a reduction of SBP itself decreases the probability of suffering a cardiac complication just after three years. Consistent with Figure 5, cardiovascular improvement is associated with an SBP intervention after this period has past, as indicated by the positive $\hat{d}(t)$ (red line).

In conclusion, the reduced risk of cardiovascular complications is primarily due to the accompanying reduction in DBP from an SBP intervention, with SBP itself having a positive impact only in the long term. This analysis demonstrates the practical performance of CSEs, showing that our algorithm’s results closely align with established findings in the literature.

It is important to note that while Causal Survival Forests are designed to estimate conditional treatment effects, they can recover marginal effects only by averaging over covariates, making them less suitable for directly estimating marginal effects. Additionally, the additive model assumption often used in the theoretical analysis of random forests^62^ may not hold in complex settings like the SPRINT trial, potentially limiting their practical utility.

## 7. Discussion

The main contribution of this paper is the introduction of a novel framework that enables model-free counterfactual inference in survival analysis, opening the doors to many tasks including uncertainty quantification and hypothesis testing. The key advantage of our method is its model-free nature, allowing for learning of complex non-linear relationships between predictors and response variables, given certain smoothness and moment conditions. Many existing models in counterfactual inference are semi-parametric in nature, like the Cox model, which may involve parameters that do not have a causally valid interpretation.^11^ The adaptation of such estimators to the fully non-parametric context faces technical difficulties, as seen with the k-NN algorithm and Beran’s estimator. However, by adopting the kernel embedding toolset, we can create model-free estimators without technical difficulties.

From a theoretical standpoint, let us discuss the implications of using weights involving just marginal Kaplan–Meier estimators. Roughly speaking, these weights assume independence between survival and censoring times, as well as conditional independence of the censoring indicator and the covariates given the realized times (Assumption v.). Let us briefly depict the consequences of relaxing these hypotheses. A regular estimator is efficient if it achieves the lowest possible variance among regular estimators, and this optimality notion is established with tools from semiparametric inference.^71^ Specifically, the Kaplan–Meier integral is asymptotically efficient only under the assumption of independence between survival and censoring times with respect to the covariates.^52,72^ This is intuitive because the covariate values of the censored times are never observed in empirical estimates. However, if we relax this hypothesis and consider a scenario where C is not independent of T given Z , or δ is not independent of X given T and Z , then the resulting estimator will be inefficient; as these assumptions were guaranteeing that the conditional survival distribution of the censoring times G does not depend on the covariates.

To address this issue, we could use a Cox model to estimate $G_{0}(t,x)$ . This would be more efficient than using Kaplan–Meier under conscious violation of the previous assumptions, but even this approach will never achieve full efficiency. As per adaptive estimation principle,^73^ a larger censoring model leads to more efficient weights estimation. However, in high-dimensional settings- the scenario we often face when covariates are present in biomedicine- the performance of this method may be poor. This may be potentially alleviated by doubly robust estimators.^74^ Our method could also be locally fitted using the “nearest neighbors” paradigm^75^ and remain robust to different types of censoring mechanisms. In summary, relaxing our hypothesis towards conditionally independent censoring would imply computing an estimator ${\hat{G}}_{z}(t,x)$ of the conditional censoring function P(C>t∣T=t,X=x,Z=z) and then replace $\frac{\Delta_{i}}{{\hat{G}}_{z}(T_{i}^{*})}$ by $\frac{\Delta_{i}}{{\hat{G}}_{z}(T_{i}^{*},X_{i})}$ for 1≤i≤n .

We utilized a RKHS approach for estimating counterfactual survival distributions with censored outcomes, leveraging the benefits of inverse censoring probability weighting due to its established history and compatibility with our chosen framework. Regarding potential inaccuracies linked to censoring distribution estimation, it is worth noting that certain techniques, such as targeted maximum likelihood (TMLE),^76^ have gained traction in recent studies addressing similar problems.^72,77^ Future explorations might delve into the integration of TMLE with our framework, refining CSEs with a targeting step and providing a comparative assessment.

The proportional hazards model is the prevailing regression model used in survival analysis. However, a standard Cox analysis does not provide insight into how the effects evolve over time, potentially resulting in loss of valuable information. With the usual Cox analysis, coefficients are typically assumed to remain constant over time, making it challenging to incorporate any deviations from this assumption. There exist a number of alternatives, for instance Aalen’s additive regression model.^78^ It offers the benefit of permitting covariate effects to vary independently over time. However, Aalen’s model performs repeated regressions at each event time, running into instability and overfitting problems when not many events (understood as uncensored observations) are present in the data. Figure 5 illustrates the importance of our estimator as a tool to assess relative risk between treatment arms across time in a natural way without involving time-dependent hazard ratios. All being said, reliably answering inferential questions about time-varying causal effects is a true milestone in contemporary statistics, even reaching areas like Reinforcement Learning.^79^

In conclusion, our proposed estimator offers a flexible and powerful tool for estimating counterfactual distributions in observational studies with right-censored data. The model-free nature of our approach makes it applicable to diverse scenarios where traditional methods may be unsuitable. Our estimator can be used in combination with or as an alternative to existing parametric and semiparametric causal survival models, further expanding the range of options available for researchers and practitioners.

## Supplemental Material

sj-zip-1-smm-10.1177_09622802241311455 - Supplemental material for Causal survival embeddings: Non-parametric counterfactual inference under right-censoring Supplemental material, sj-zip-1-smm-10.1177_09622802241311455 for Causal survival embeddings: Non-parametric counterfactual inference under right-censoring by Carlos García Meixide and Marcos Matabuena in Medical Research

sj-pdf-2-smm-10.1177_09622802241311455 - Supplemental material for Causal survival embeddings: Non-parametric counterfactual inference under right-censoring Supplemental material, sj-pdf-2-smm-10.1177_09622802241311455 for Causal survival embeddings: Non-parametric counterfactual inference under right-censoring by Carlos García Meixide and Marcos Matabuena in Medical Research
