# Supplementary material for: Causal survival embeddings: Non-parametric counterfactual inference under right-censoring
Source: Stat Methods Med Res. 2025 Feb 11;34(3):574–93. doi: 10.1177/09622802241311455 (PMC11951469; doi:10.1177/09622802241311455)
Supplement: sj-pdf-2-smm-10.1177_09622802241311455 - Supplemental material for Causal survival embeddings: Non-parametric counterfactual inference under right-censoring [file sj-pdf-2-smm-10.1177_09622802241311455.pdf]

Supplement: Causal survival embeddings: non-parametric counterfactual inference under right-censoring

1 Extended background

Treatment effect estimation using survival endpoints is of key interest in statistical and biomedical applications. However, the processes structuring clinical research over the last decades tend to preclude collecting data arising from the optimal study design regarding a particular medical question of interest. As a consequence, observational studies are more and more present in scientific research due to technical limitations that render impossible randomization- the gold standard experimental design practice to protect against unmeasured confounding. One key factor is the growing complexity of modern healthcare and medical interventions. In many cases, it is not ethically feasible or practically possible to randomize patients into different treatment groups. Additionally, as medical treatments become more personalized and tailored to individual patients, the traditional randomized controlled trial (RCT) design may not capture the full spectrum of patient responses. Furthermore, observational studies allow researchers to study the effects of treatments or interventions in real-world settings, providing insights into how they perform under diverse conditions. They offer the opportunity to examine long-term outcomes, rare events, and patient populations that might be excluded from RCTs.

While RCTs remain the gold standard for establishing causality, observational studies offer insights into real-world scenarios that are not achievable through controlled experiments. Advances in data collection, and the integration of electronic health records have also contributed to the increased utilization of observational studies in clinical research. As clinical research adapts to these evolving challenges and seeks insights beyond traditional randomized study designs, it is crucial to address potential pitfalls in interpreting treatment effects accurately. Notably, extensive warnings have surfaced in the literature concerning causal interpretations of hazard ratios (HR) estimated with Cox (1972)'s Pro-

portional Hazards (PH) model, even under randomized treatment exposures (Hernán 2010, Stensrud et al. 2018). As a matter of fact, HRs merge at each instant the differences between arms that arise from treatment effect with those created by selection bias: intuitively, as time goes by, less patients will remain in the control arm if overall mortality risk is different between both groups (equivalently, when the treatment is effective) leading to a comparison between unbalanced groups.

This makes clear the point that there is a need for designing new effect measures within survival analysis that have a causal interpretation and shed light into time dynamics, for instance time-varying treatment effects. Assume that gynaecologists aim to investigate the effect of an implanted medical device, such as a contraceptive method, on time-to-conception. It is reasonable to consider that the implant gradually deteriorates and it will cease to function as time goes by. Martinussen (2022) has shown that a Cox model fails in this setup despite participants being randomized.

Causal treatment effect assertions are mathematically formalized via the potential outcome framework, the fundamental paradigm for statistical analysis of observational data - where treatment is not independent of the covariates (Neyman 1923, Rubin 1974). Numerous studies center their focus on calculating the average treatment effect (ATE, see Imbens (2004)), which quantifies the difference between the means of outcome distributions. This parameter is particularly significant in observational studies because the ATE takes on an interpretation aligned with causal inference principles within a framework described by Pearl et al. (2000). It represents the outcome that would have been observed had the treatment been allocated randomly, offering a means to disentangle the effects of confounding variables.

Most empirical research on treatment effects typically focuses on estimating mean differences, which provide a clear and interpretable measure of the average effect a treatment

has on a particular outcome across different groups. This information is crucial for both clinical decision-making and policy formulation. Overall, estimating mean differences is a cornerstone of treatment effect research as it provides a concise and informative summary of the impact of interventions.

However, there is also a longstanding interest in developing methods to estimate the impact of treatments on the entire outcome distribution. For example, if a specific treatment's impact is only observed in the outcome distribution's variance (Park et al. 2021), the evaluation of average treatment effects will not be informative in clinical decision-making because only first order discrepancies between treatment arms would be captured. A straight generalization would be to focus on the difference between the survival functions of potential outcomes directly on the absolute scale, as it is a causally meaningful quantity that does not rely on non-collapsible parameters (Aalen et al. 2015) nor quantities whose identifiability relies on unstable assumptions (see Section 5 in Martinussen (2022)).

Estimating potential outcome distributions directly is straightforward when treatment assignment is random. For instance, under right-censoring it would suffice to fit one Kaplan & Meier (1958) curve per arm. However, in observational studies (or randomized experiments with imperfect compliance), capturing distributional differences between two groups becomes challenging (Imbens & Rubin 1997). In this line, distributional extensions of the ATE have been considered in the literature through multiple lenses. For example, Abadie (2002) considers a bootstrap strategy to estimate distributional treatment effects while Muandet, Kanagawa, Saengkyongam & Marukatat (2021) base their work on the theory of reproducing kernel Hilbert spaces (RKHSs).

When data was not generated through an RCT, the methodology tackling estimation of treatment effects can be clustered in two families. On the one hand, estimators relying on the imputation of the so-called propensity scores- which are the conditional probabilities

of treatment assignment given the covariates (Rosenbaum & Rubin 1983). The fundamental technique here is named *inverse probability of treatment weighting* estimation (Imbens 2004), consisting loosely of performing an empirical inner product between the summands of the unweighted estimator times the reciprocals of the estimated propensity scores. It basically involves reweighing observations based on the propensity score, following the Horvitz & Thompson (1952) approach.

On the other hand, significant efforts have been made pursuing alternative procedures to avoid weighting in treatment effect estimation. Those consist of estimating counterfactual distributions based on regressing conditional distributions (Chernozhukov et al. 2013). Counterfactual analysis is crucial in other research areas such as empirical economics (Juhn et al. 1993, Stock 1989), although it also plays a core role in medicine (Robins & Greenland 1989). In this field, it helps by simulating what would have happened if everyone received a new treatment, allowing researchers to estimate its true effect while accounting for these differences. This approach provides insights into the treatment's effectiveness in real-world settings. A counterfactual survival function $S_{\langle j|k \rangle}$ equals one minus the distribution function of a time-to-event response variable created by first sampling a value from the covariate distribution of group k and then sampling from the conditional distribution of group j plugging in the previously obtained covariate value.

Under correct model specification, both weighting and regression lenses hold equal validity. Notably, if we saturate the specification of the propensity score and conditional distribution models, both methods yield numerically identical outcomes. Traditional methods for estimating the latter involve parametric approaches like logistic regression, which rely on a model for treatment propensity. However, incorrect specifications of the model can generate extreme weights and make the estimator unreliable Ma & Wang (2020). To address this issue, nonparametric techniques have been proposed (Lee et al. 2010). Nonetheless,

large weights may still be unavoidable even when the propensity score model is correctly specified. The regression approach also holds clear interpretation advantages with respect to weighting. As a matter of fact, estimation of the conditional model constitutes itself independent interest in medicine (Cox 1972).

In addition, counterfactual distributions allow to decouple the difference between *realized outcome*- not potential- survival distributions in a particular form inherited from the econometrics literature (Oaxaca 1973, Blinder 1973). This decomposition strategy involves two terms: one driven by shifts in the covariate distributions between groups and other accounting for the distributional treatment effect conditional to the treated arm. This provides a formal mechanism to analyze whether differences that arise from the observed outcomes distributions of each arm truly come from effectiveness of a drug, whether they are just due to the probabilistic structure of the population baseline characteristics, or both.

Estimating the elements present in the decomposition mentioned above provides the primary motivation for our notion of *causal survival embedding*. The main intuition behind the idea is that these objects allow for further investigation of what mechanism is the origin for potential differences that arise between the observational survival functions of two treatment arms (i.e. Kaplan-Meier curves fitted to isolated data coming from each treatment indicator value).

2 Tutorial overview

Let us illustrate the impact of our framework by checking the insights it can provide when applied to two simulation examples, Case I and Case II. We encode both mechanisms with the following model, generating n and m individuals in control and treatment arms,

respectively. `T0tilde` and `T1tilde` are each $n + m$ counterfactual log-times (in practice, we only have access to one of the two, dictated by treatment indicator) and `X0` and `X1` are the covariate observations for individuals in the control and treatment groups, respectively.

```
T0tilde=rnorm(n+m,mean=b0[1]*X[,1]+b1[2]*X[,2],sd=sd0)
T1tilde=rnorm(n+m,mean=b1[1]*X[,1]+b1[2]*X[,2]+shift_time,sd=sd1)

X0=cbind(rnorm(n),rnorm(n))
X1=cbind(rnorm(m)+shift_cov,rnorm(m))
X=rbind(X0,X1)
```

In Case I, the divergence between the observational Kaplan-Meier estimators is driven by the treatment itself. This discrepancy arises due to distinct conditional probability laws of time-to-event between both groups, also resulting in disparate counterfactual time distributions. This scenario provides insights into setups where the treatment itself affects the outcome distributions, necessitating precise counterfactual estimations to disentangle the true treatment effects from underlying confounding factors. It is important to note that the distribution of covariates remains the same across both groups, reflecting an scenario where treatment would be proven to be effective in practice. We reproduce this setting in our simulation model with the following configuration: `shift_cov` $= 0$, `shift_time` $\neq 0$, `sd0` $\neq$ `sd1` and `b0` $\neq$ `b1`

The upper-left plot showcases the treatment group time densities with blue lines and the control group with black lines. It is important to recognize that the dotted lines, representing the counterfactual distributions, are unobservable in practice. Consequently, we can only estimate the distributions arising from integrating the solid lines, which depict the

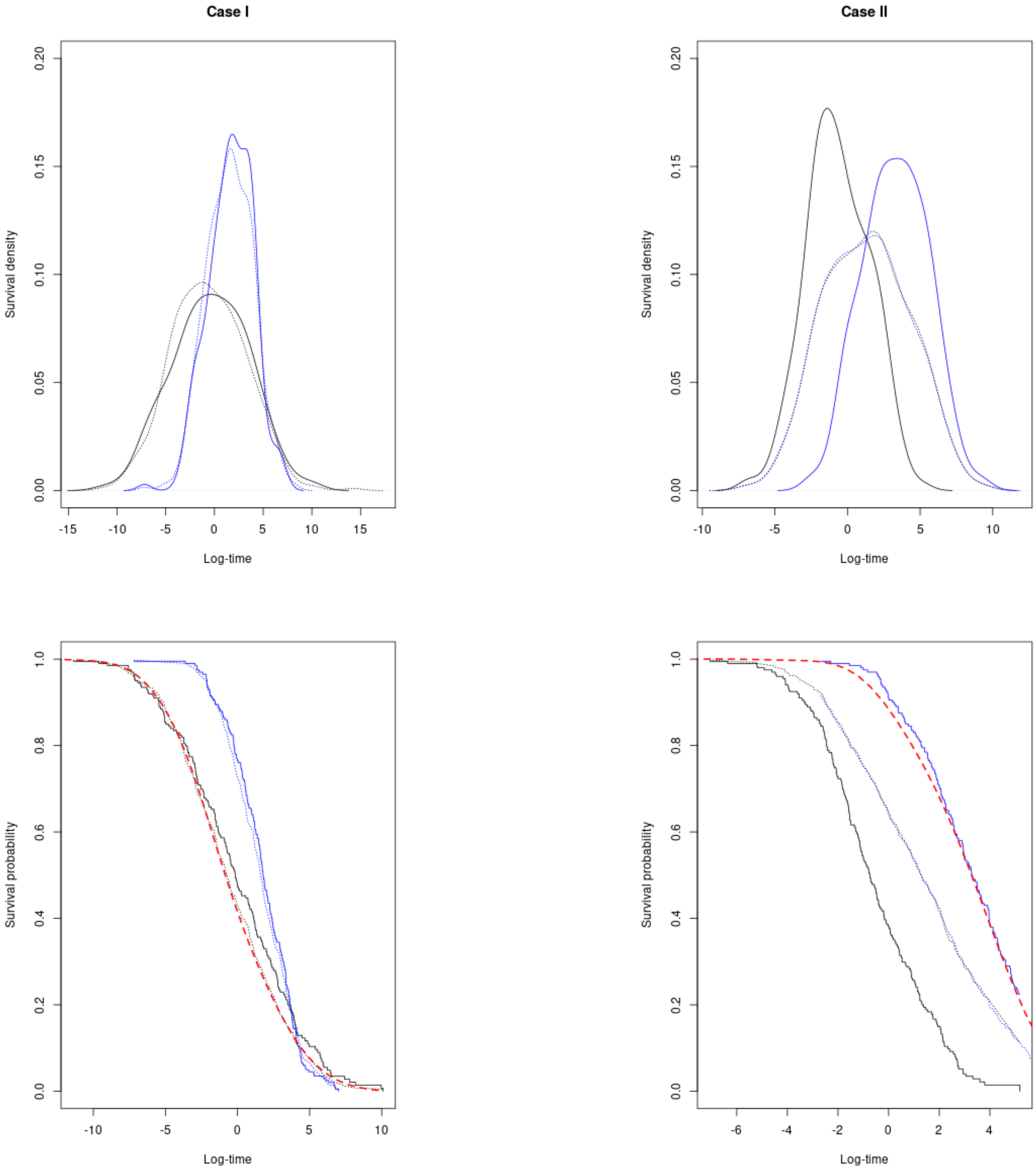

(a) (b)

Figure 1: Main illustration of the impact brought by causal survival embeddings

distributions that conditional to each group calculated just by isolating the data collected in each arm. In absence of confounding, the counterfactual distributions coincide with the conditional distributions due to the treatment's genuine impact on survival times and subsequent alterations in potential outcomes.

Moving to the lower-left depiction, we observe the same mechanism represented through survival functions. Here, the blue Kaplan-Meier curves signify the treatment arm, while the black curves represent the control group. It is worth emphasizing that the counterfactuals align with the conditionals due to the absence of any association between treatment indicator and covariates. Our methodology yields the red dashed line, an estimator of $S_{\langle 0|1\rangle}$ in

$$S_{\langle 1|1\rangle} - S_{\langle 0|0\rangle} = \left[S_{\langle 1|1\rangle} - S_{\langle 0|1\rangle}\right] + \left[S_{\langle 0|1\rangle} - S_{\langle 0|0\rangle}\right]. \tag{1}$$

By assessing its proximity to the solid black and blue lines, representing $S_{\langle 0|0\rangle}$ and $S_{\langle 1|1\rangle}$ respectively, we can quantify selection bias. Being the red line close to the black one signifies that the second discrepancy on the right hand side is zero, and consequently, the observed difference between the Kaplan-Meier curves, calculated by isolating the data from each group, is a result of the treatment's genuine impact, also causing divergent potential outcomes.

Next, we explore Case II, where the conditional distribution of times remains the same across both groups, but a disparity in covariate distribution emerges. This setting reflects the real-world scenario where treatment effects may be confounded by variations in patient characteristics (think of surgery as treatment and in a clinical setup where younger people are more likely to receive surgery). We model this scenario by setting `shift_cov` $\neq 0$, EDIT `shift_time` $= 0$, `sd0` $=$ `sd1` and `b0` $=$ `b1`

In the top-right plot, we observe different observational density functions while the counterfactual ones remain identical. This disparity arises due to the absence of any actual

effect resulting from the treatment, with survival times differing solely due to a shift in the distribution of covariates.

In the bottom-right depiction, once again returning to the realm of survival functions, we notice that the counterfactual Kaplan-Meier curves practically coincide, but remain unattainable in practicality as we can only observe one potential outcome per individual. The empirically estimated curves differ due to structural dissimilarities in the distribution of covariates. At this point, our estimator closely aligns with the blue Kaplan-Meier curve. This suggests that confounding factors significantly contribute to survival observational difference between arms since the first difference on the right hand side of the decomposition is nearly zero.

3 More on vector-valued RKHSs

We start by noticing that the map $x \mapsto \mu_{T^0|X^0=x}$ takes values in the Hilbert space $\mathcal{H}$. This motivates the following definition (see for example Carmeli et al. (2006))

Definition 1 (Vector-valued RKHS). *An $\mathcal{H}$-valued RKHS on $\mathcal{X}$ is a Hilbert space $\mathcal{F}$ such that 1) the elements of $\mathcal{F}$ are functions $\mathcal{X} \to \mathcal{H}$; 2) for all $x \in \mathcal{X}, \exists C_x > 0$ such that $\|F(x)\|_{\mathcal{H}} \leq C_x \|F\|_{\mathcal{F}}$ for all $F \in \mathcal{F}$.*

In the traditional framework of RKHSs formed by real valued functions, a very useful aspect is that it is possible to evaluate functions belonging to the space by making inner products times very special elements therein: the collection $\{k(\cdot, x) : x \in \mathcal{X}\}$. k is the so-called *kernel* function uniquely determining $\mathcal{H}$. Looking for a surrogate of the notion of kernel in $\mathcal{H}$-valued RKHSs we arrive to the following definition. We call $\mathcal{L}(\mathcal{H})$ the space of bounded linear operators from $\mathcal{H}$ to $\mathcal{H}$.

Definition 2 ($\mathcal{H}$-kernel)**.** *A $\mathcal{H}$-kernel of positive type on $\mathcal{X}\times\mathcal{X}$ is a map $\Gamma : \mathcal{X}\times\mathcal{X} \to \mathcal{L}(\mathcal{H})$ such that $\forall N \in \mathbb{N}, \forall x_1,\ldots,x_N \in \mathcal{X}$ and $\forall c_1,\ldots,c_N \in \mathbb{R}, \sum_{i,j=1}^{N} c_i c_j \langle \Gamma(x_j, x_i)(h), h\rangle_{\mathcal{H}} \geq 0 \quad \forall h \in \mathcal{H}$.*

If Γ is an $\mathcal{H}$-kernel in the sense of the previous definition, there exists a unique (up to isometry) RKHS, with Γ as its reproducing kernel (Micchelli & Pontil 2005), satisfying: 1) for any $x, x' \in \mathcal{X}, h, h' \in \mathcal{H}$ and $F \in \mathcal{F}$, $\langle F(x), h\rangle_{\mathcal{H}} = \langle F, \Gamma(\cdot, x)(h)\rangle_{\mathcal{F}}$ and 2) $\langle h, \Gamma(x, x')(h')\rangle_{\mathcal{H}} = \langle \Gamma(\cdot, x)(h), \Gamma(\cdot, x')(h')\rangle_{\mathcal{F}}$

4 Counterfactual distributions from estimators of their kernel mean embedding

Let $F_{T\langle 0|1\rangle}$ and $F_{T\langle 1|0\rangle}$ be counterfactual distributions. For simplicity, let us consider the first one (what follows also holds for the second one). Kanagawa & Fukumizu (2014) introduce a general framework to recover distributions from Gaussian RKHS embeddings implying that $F_{T\langle 0|1\rangle}$ can be estimated from $\hat{\mu}_{T\langle 0|1\rangle}$ without any parametric assumptions on $F_{T\langle 0|1\rangle}$.

From the expression for the causal survival embedding in Section 3, we can see that in reality we are estimating the kernel mean embedding of $F_{T\langle 0|1\rangle}$ with samples of $F_0^{(*)}$ and therefore it has a form of weighted sum of feature vectors (following Song et al. (2013)): $\hat{\mu}_{T\langle 0|1\rangle} := \sum_{i=1}^{n} w_i l(\cdot, T_i^*)$ with weights $w_1,\ldots,w_n \in \mathbb{R}$ being the entries of the vector $1_m'\tilde{K}'(WK + n\varepsilon I)^{-1}W$.

Let P be the probability measure associated with $F_{T\langle 0|1\rangle}$ and Q that associated with $F_0^{(*)}$. Kanagawa & Fukumizu (2014) show that $P(\Omega)$ on any interval $\Omega \subset (0, +\infty)$ may be estimated from $\hat{\mu}_{T\langle 0|1\rangle}$. Here, we define an interval in $(0, +\infty)$ as $\Omega := [a, b]$. An estimator of

$P(\Omega)$ is defined by the sum of weights of points in Ω, i.e. $\hat{P}(\Omega) = \sum_{T_i^* \in \Omega} w_i$. The following theorem provides its convergence rate on expectation.

Theorem 1 (Kanagawa & Fukumizu (2014)). *Assume that $F_{T\langle 0|1\rangle}$ and $F_0^{(*)}$ have densities that belong to $L_\infty(0, +\infty)$, and that l is a Gaussian kernel. Assume that $\hat{\mu}_{T\langle 0|1\rangle}$ is consistent and that $\mathbb{E}\left[\left\|\hat{\mu}_{T\langle 0|1\rangle} - \mu_{T\langle 0|1\rangle}\right\|_{\mathcal{H}}\right] = O\left(n^{-b}\right)$ and $\mathbb{E}\left[\sum_{i=1}^n w_i^2\right] = O\left(n^{-2c}\right)$ for some $0 < b, c \leq 1/2$ as $n \to \infty$. In addition, assume that $b - d(1-2c) > 0$. Then for arbitrary small $\xi > 0$, we have*

$$\mathbb{E}\left[\left|\sum_{T_i^* \in \Omega} w_i - P(\Omega)\right|\right] = O\left(n^{-\frac{b-d(1-2c)}{1+2d}+\xi}\right).$$

5 Proof for convergence rate of the stochastic error

Proof. We start with the same breakdown as in proof of Theorem 11 in Fukumizu, Song & Gretton (2013):

$$\begin{aligned}
&\left\|\widehat{\mathcal{C}^*}_{TX}\left(\widehat{\mathcal{C}^*}_{XX} + \varepsilon_n I\right)^{-1}\widehat{\mu}_{X_1} - \mathcal{C}_{TX}\left(\mathcal{C}_{XX} + \varepsilon_n I\right)^{-1}\mu_{X_1}\right\|_{\mathcal{H}} \leq \\
&\leq \underbrace{\left\|\widehat{\mathcal{C}^*}_{TX}\left(\widehat{\mathcal{C}}_{XX} + \varepsilon_n I\right)^{-1}\left(\widehat{\mu}_{X_1} - \mu_{X_1}\right)\right\|_{\mathcal{H}}}_{(A)} \\
&+ \underbrace{\left\|\left(\widehat{\mathcal{C}^*}_{TX} - \mathcal{C}_{TX}\right)\left(\mathcal{C}_{XX} + \varepsilon_n I\right)^{-1}\mu_{X_1}\right\|_{\mathcal{H}}}_{(B)} \\
&+ \underbrace{\left\|\widehat{\mathcal{C}^*}_{TX}\left(\widehat{\mathcal{C}^*}_{XX} + \varepsilon_n I\right)^{-1}\left(\mathcal{C}_{XX} - \widehat{\mathcal{C}^*}_{XX}\right)\left(\mathcal{C}_{XX} + \varepsilon_n I\right)^{-1}\mu_{X_1}\right\|_{\mathcal{H}}}_{(C)}
\end{aligned}$$

(A): From Muandet et al. (2021) we have that

$$\text{(A)} = O_p\left(\varepsilon_n^{-1/2} n^{-1/2}\right)$$

as it can be seen to rely on weak convergence of uncensored kernel mean embeddings at speed $\frac{1}{\sqrt{n}}$ (Ledoux & Talagrand 1991, Berlinet & Thomas-Agnan 2011) and on applying Theorem 1 in Baker (1973) to $\frac{d\hat{F}_0^{(*)}}{\hat{G}_0}$.

(B): using Lemma 24 in Muandet et al. (2021)

$$\begin{aligned}\left\|\left(\widehat{\mathcal{C}^*}_{TX} - \mathcal{C}_{TX}\right)\left(\mathcal{C}_{XX} + \varepsilon_n I\right)^{-1}\mu_{X_1}\right\|_{\mathcal{H}} &\leq \left\|\widehat{\mathcal{C}^*}_{TX} - \mathcal{C}_{TX}\right\| \left\|\left(\mathcal{C}_{XX} + \varepsilon_n I\right)^{-1}\mu_{X_1}\right\|_{\mathcal{G}} \\ &\leq \left\|\widehat{\mathcal{C}^*}_{TX} - \mathcal{C}_{TX}\right\| \cdot O_p\left(\varepsilon_n^{-1/2}\right)\end{aligned}$$

(C): proceeding as in Muandet et al. (2021)

$$\text{(C)} = \left\|\widehat{\mathcal{C}}^*_{XX} - \mathcal{C}_{XX}\right\| \cdot O_p\left(\varepsilon_n^{-1}\right)$$

Let $\varepsilon_n > 0$ be a regularization constant. Then if $\varepsilon_n \to 0$ and $n^{1/2}\varepsilon_n \to \infty$ as $n \to \infty$, we have consistency provided that we show the tight uniform bounds

$$\left\|\widehat{\mathcal{C}^*}_{XX} - \mathcal{C}_{XX}\right\| = O_p\left(n^{-1/2}\right) \tag{2}$$

$$\left\|\widehat{\mathcal{C}^*}_{TX} - \mathcal{C}_{TX}\right\| = O_p\left(n^{-1/2}\right) \tag{3}$$

and the term with the the slowest rate would be (C). We will have into account that $\|\cdot\| \leq \|\cdot\|_{HS}$.

In the following, $\mathcal{G} \otimes \mathcal{H}$ denotes the direct product of $\mathcal{G}$ and $\mathcal{H}$ (see for example Reed & Simon (1980))

Lemma 1. *Define* $K_i = k(\cdot, X_i) - \mu_{X^0}$*,* $L_i = l(\cdot, T_i^*) - \mu_{T^0}$*,* $K(X^0) = k(\cdot, X^0) - \mu_{X^0}$*,* $L(T^0) = l(\cdot, T^0) - \mu_{T^0}$ *where* μ_{X^0} *and* μ_{T^0} *are the marginal kernel mean embeddings* $E_{X^0}\left[k(\cdot, X^0)\right]$ *and* $E_{T^0}\left[k(\cdot, T^0)\right]$*. Then we have:*

$$\|\widehat{\mathcal{C}^*}_{TX} - \mathcal{C}_{TX}\|_{HS}^2 = \left\| \frac{1}{n}\sum_{i=1}^n W_i \left(K_i - \frac{1}{n}\sum_{j=1}^n W_j K_j \right) \left(L_i - \frac{1}{n}\sum_{j=1}^n W_j L_j \right) - E[K(X^0)L(T^0)] \right\|_{\mathcal{G}\otimes\mathcal{H}}^2$$

Proof. Direct adaptation of Fukumizu et al. (2007). □

Deriving the following inequality in Lemma 2 is more involved.

Lemma 2.

$$\|\widehat{\mathcal{C}^*}_{TX} - \mathcal{C}_{TX}\|_{HS} \le$$

$$\le \left\| \frac{1}{n}\sum_{i=1}^n W_i K_i L_i - E[K(X^0)L(T^0)] \right\|_{\mathcal{G}\otimes\mathcal{H}} + \left| 2 - \frac{1}{n}\sum_{i=1}^n W_i \right| \left\| \frac{1}{n}\sum_{i=1}^n W_i K_i \right\|_{\mathcal{G}} \left\| \frac{1}{n}\sum_{i=1}^n W_i L_i \right\|_{\mathcal{H}}$$

Proof. See Section 9. □

Let us denote for simplicity of notation $\mu_{X^0} = \mu_0$. Having a closer look at the term $\left\|\frac{1}{n}\sum_{i=1}^n W_i K_i\right\|_{\mathcal{G}}$ on the right hand side of Lemma 2

$$\begin{aligned}
\frac{1}{n}\sum_{i=1}^n W_i K_i = \frac{1}{n}\sum_{i=1}^n W_i (k(\cdot, X_i) - \mu_0) &= \frac{1}{n}\sum_{i=1}^n (W_i k(\cdot, X_i) - W_i \mu_0) \\
&= \frac{1}{n}\sum_{i=1}^n W_i k(\cdot, X_i) - \mu_0 + \mu_0 - \mu_0 \left(\frac{1}{n}\sum_{i=1}^n W_i \right) = \\
&= \left(\frac{1}{n}\sum_{i=1}^n W_i k(\cdot, X_i) - \mu_0 \right) + \mu_0 \left(1 - \frac{1}{n}\sum_{i=1}^n W_i \right)
\end{aligned}$$

Furthermore,

$$\frac{1}{n}\sum_{i=1}^n W_i k(\cdot, X_i) - \mu_0 = \int_{\mathcal{X}} k(\cdot, X^0) \frac{d\hat{F}_0^{(*)}}{\hat{G}_0} - \int_{\mathcal{X}} k(\cdot, X^0) \frac{dF_0^{(*)}}{G_0} =: \nu(\hat{F}_0^{(*)}, \hat{G}_0) - \nu(F_0^{(*)}, G_0) \in \mathcal{G}$$

It is important to note that ν is an operator taking values in a Hilbert space and showing its Hadamard-differentiability is not straightforward. Let, for $n \geq 1$, $S_n = \sum_{i=1}^{n} k(\cdot, X_i)$ and $\Lambda_n = \sqrt{n}\left(\frac{S_n}{n} - \mathcal{I}_\mu\right)$. Since $\mathcal{I}_\mu = \int K(\cdot, X_0)\, dF_{X^0} = E\left(K(\cdot, X_0)\right)$, one could prove by using the Hilbert space version of the Central Limit Theorem that the sequence $(\Lambda_n)_{n\geq 1}$ converges weakly to a centered Gaussian variable (Ledoux & Talagrand 1991). However, we cannot proceed this way because the W_i are breaking the i.i.d. assumption needed by this CLT.

We now proceed to show Hadamard-differentiability of ν for $\mathcal{X} = \mathbb{R}$. The following definitions are taken from Van der Vaart (2000) sections 18.6 and 20.3

Definition 3. *Let $T = [a, b]$ be an interval in the extended real line. We denote by $C[a, b]$ the set of all continuous functions $z : [a, b] \mapsto \mathbb{R}$ and by $D[a, b]$ the set of all functions $z : [a, b] \mapsto \mathbb{R}$ that are right continuous and whose limits from the left exist everywhere in $[a, b]$. (The functions in $D[a, b]$ are called cadlag: continue à droite, limites à gauche.)*

The space $D[a, b]$ is referred to here as the Skorohod space. It can be shown that $C[a, b] \subset D[a, b] \subset \ell^\infty[a, b]$. We always equip the spaces $C[a, b]$ and $D[a, b]$ with the uniform norm $\|z\|_T$, which they "inherit" from $\ell^\infty[a, b]$.

The set $BV_M[a, b]$ is the set of all cadlag functions $z : [a, b] \mapsto [-M, M] \subset \mathbb{R}$ of variation bounded by M. We also define:

$BV_M^1[a, b] = \{B \in BV_M[a, b] : x \mapsto k(x, x) \in L^1(B)\}$

$D^2[a, b] = \{A \in D[a, b] : A \in L^2(B) \text{ for all } B \in BV_M^1[a, b]\}$

We need to restrict our operator to $D_M \equiv D^2[-\infty, \infty] \times BV_M^1[-\infty, \infty]$ for existence of Bochner integrals, see Theorem 105 in Berlinet & Thomas-Agnan (2011). Nevertheless, thanks to assumptions i.) and vi.) this is always the case as far as we operate on $D[a, b] \times BV_M[a, b]$.

Before proving the following lemma, it is useful to make a remark on assumption 1, which helps in conjunction with the following general inequality for RKHSs. Let us suppose that $f \in \mathcal{G}$. Then for $x \in \mathcal{X}$

$$f(x) = \langle k(\cdot, x), f \rangle_{\mathcal{H}} \leq \|k(\cdot, x)\|_{\mathcal{H}} \|f\|_{\mathcal{H}}$$

by Cauchy-Schwartz inequality. Noting that $\|k(\cdot, x)\|_{\mathcal{H}}^2 = \langle k(\cdot, x), k(\cdot, x) \rangle_{\mathcal{H}} = k(x, x)$, we finally have

$$f(x) \leq \sqrt{k(x, x)} \|f\|_{\mathcal{H}}$$

and therefore

$$\|f\|_{\infty} \leq \sup_{x \in \mathcal{X}} |k(x, x)| \|f\|_{\mathcal{H}}$$

In particular,

$$k(x, x') \leq \sqrt{k(x, x)} \sqrt{k(x', x')}$$

Moreover, as all probability measures are finite we have ensured that k is integrable with respect to any probability measure due to Hölder's inequality.

Lemma 3. *Let $\mathcal{H}$ be an RKHS of functions $f : \mathbb{R} \longrightarrow \mathbb{R}$ with reproducing kernel k. Then the operator $(A, B) \mapsto \int k(\cdot, x) A(x) dB(x) \in \mathcal{H}$ is Hadamard-differentiable from the domain $D_M \equiv D^2[-\infty, \infty] \times BV_M^1[-\infty, \infty] \subset D[-\infty, \infty] \times D[-\infty, \infty]$ into $(\mathcal{H}, \sqrt{\langle \cdot, \cdot \rangle_{\mathcal{H}}})$ at every pair of functions of bounded variation (A, B).*

Proof. We set as a candidate $\psi'_{A,B}(\alpha, \beta)(\cdot) = \int k(\cdot, x) A(x) d\beta(x) + \int k(\cdot, x) \alpha(x) dB(x)$, for $(\alpha, \beta) \in D_M$ and use the fact that $\|k(\cdot, x)\|_{\mathcal{H}}^2 = \langle k(\cdot, x), k(\cdot, x) \rangle = k(x, x)$.

For sequences $t_n \to 0$ in $\mathbb{R}$, $\alpha_n \to \alpha$, and $\beta_n \to \beta$ in $D^2[-\infty, \infty]$ and $BV_M^1[-\infty, \infty]$ respectively, define $A_n \equiv A + t_n\alpha_n$ and $B_n \equiv B + t_n\beta_n$. Since we require that $(A_n, B_n) \in D_M$, we know that the total variation of B_n is bounded by M. Consider first the derivative of ψ, and note that

$$\begin{aligned}
&\left\| \frac{\int k(\cdot, x)A_n(x)dB_n(x) - \int k(\cdot, x)A(x)dB(x)}{t_n} - \psi'_{A,B}\left(\alpha_n, \beta_n\right) \right\|_{\mathcal{H}} = \\
&\left\| \int k(\cdot, x)\alpha_n(x)d\left(B_n - B\right)(x) \right\|_{\mathcal{H}} = \\
&\left\| \int k(\cdot, x)\alpha(x)d\left(B_n - B\right)(x) + \int k(\cdot, x)\left(\alpha_n(x) - \alpha(x)\right)d\left(B_n - B\right)(x) \right\|_{\mathcal{H}} \leq \\
&\int \|k(\cdot, x)\|_{\mathcal{H}}\,|\alpha(x)|d\left(B_n - B\right)(x) + \int \|k(\cdot, x)\|_{\mathcal{H}}\,|\alpha_n(x) - \alpha(x)|d\left(B_n - B\right)(x) = \\
&\underbrace{\int \sqrt{k(x, x)}|\alpha(x)|d\left(B_n - B\right)(x)}_{(1)} + \underbrace{\int \sqrt{k(x, x)}\left(|\alpha_n(x) - \alpha(x)|\right)d\left(B_n - B\right)(x)}_{(2)}
\end{aligned}$$

Because we assumed that k is bounded, (2) converges to zero as since both B_n and B have total variation bounded by M and k is bounded. For convergence of (1) to zero as $t_n \longrightarrow 0$, we follow the same argument as in Van der Vaart (2000), Lemma 20.10., with ϕ therein the identity map.

Since the map $(\alpha, \beta) \mapsto \psi'_{A,B}(\alpha, \beta)$ is continuous and linear, the desired Hadamard differentiability of ψ will follow because (1) and (2) converge to zero.

□

Our operator ν was defined after Lemma 2 for $(A, B) \in D_M$ as

$$\nu : (A, B) \mapsto \left(A, \frac{1}{B}\right) \mapsto \int_{\mathcal{X}} k(\cdot, X)\frac{1}{B(X)}dA(X)$$

First, it is known that $\sqrt{n}(\hat{G}_0 - G_0)$ converges weakly in $D[0, \tau]$ to a tight, mean zero Gaussian process (Fleming & Harrington (2011), Andersen et al. (2012)). Second, by

Donsker's theorem, $\sqrt{n}(\hat{F}_0^{(*)} - F_0^{(*)})$ also converges weakly to a tight, mean zero Gaussian process. As a reminder, $d\hat{F}_0^{(*)}(t,x) = \frac{1}{n}\sum_{i=1}^n \Delta_i \delta_{T_i^*}(t)\delta_{X_i}(x)$ is just the empirical measure of the uncensored observations on the arm with $Z_i = 0$

We can assert that $\sqrt{n}\left(\nu(\hat{F}_0^{(*)}, \hat{G}_0) - \nu(F_0^{(*)}, G_0)\right)$ converges weakly to a process in a Polish RKHS due to the chain rule of Hadamard-differentiability, the fact that $B \mapsto 1/B$ is Hadamard differentiable on $\{B \in \ell^\infty(\mathcal{X}) : \inf_{x\in\mathcal{X}} |B(x)| > 0\}$, Lemma 3 and the Functional Delta Method (Kosorok 2008). Therefore, in virtue of Prokhorov's theorem the limiting process is uniformly tight and therefore:

$$\left\| \frac{1}{n}\sum_{i=1}^n W_i(k(\cdot, X_i) - \mu_{X^0}) \right\|_{\mathcal{G}} = O_p\left(n^{-1/2}\right), \quad \left\| \frac{1}{n}\sum_{i=1}^n W_i(l(\cdot, T_i^*) - \mu_{T^0}) \right\|_{\mathcal{H}} = O_p\left(n^{-1/2}\right)$$

By consistency of real Kaplan-Meier integrals (Stute 1993): $\frac{1}{n}\sum_{i=1}^n W_i = o_p(1)$. In addition, the tensor product norm in right hand side of Lemma 2 can be seen to be $O_p(n^{-1/2})$ combining our arguments with those in Lemma 5 from Fukumizu et al. (2007). By Slutsky's theorem, we have just shown the tight uniform bounds (2), (3) we were looking for. □

6 Selection of regularization parameter

In this section, we present a new approach to selecting the regularization constant ε involved in the estimation of conditional mean embeddings. Drawing inspiration from the 'match-then-split' principle (Gao et al. 2021), we devise a novel cross-validation routine tailored to our framework. At its core, we employ a sophisticated goodness-of-fit test (Fernández & Gretton 2019) designed specifically for censored data that makes no assumptions on the alternative class, enabling us to make informed decisions regarding the regularization constant. This innovative method not only enhances the robustness of causal survival embeddings but also contributes to the broader field of hyperparameter selection for complex

statistical techniques. We describe the criterion introduced by Fernández & Gretton (2019) and define $\tilde{F}$ involved in Algorithm 1. In the context of independent right-censoring, the focus is on testing the null hypothesis $H_0 : F_{T\langle 0|1\rangle} = F_0$, where F_0 is an estimated candidate. It is known that F_0 evaluated on the uncensored times-to-event follows a $\mathcal{U}(0,1)$ distribution under the null hypothesis.

In the case of right-censored data, the increasing nature of F_0 (a distribution function) allows the indicator function Δ_i to be coherent with how $F_0(T_i^*)$ and $F_0(C_i)$ are ordered. When $\Delta_i = 1$, $U_i = F_0(T_i)$ and a point mass of size $1/n$ is placed on U_i using the empirical distribution approach. Alternatively, when $\Delta_i = 0$, $U_i = F_0(C_i)$ and the only known information is $F_0(T_i) > F_0(C_i)$. Here, the weight $1/n$ associated with U_i is spread uniformly over $(U_i, 1)$. The resulting estimator $\tilde{F}$ is:

$$\tilde{F}(x) = \frac{1}{n} \sum_{U_i \leq x} \Delta_i + (1 - \Delta_i) \frac{x - U_i}{1 - U_i}.$$

Proposition 1 (Fernández & Gretton (2019)). *Under the null hypothesis, the estimator $\tilde{F}$, based on the data $\{U_i, \Delta_i\}_{i=1}^n$, is an unbiased estimator of the uniform distribution function F_{unif}.*

Notice that Algorithm 1 requires a matched dataset as an input. This set is constructed by selecting a subset of observations such that treated and control units with similar or closely aligned characteristics are paired. Let M denote the set of indices of the units in the matched dataset. Then, for any $i \in M$, there exists a corresponding matched unit $j \in M$ such that $Z_i = 1$ and $Z_j = 0$, and the covariates X_i and X_j are similar or well-matched. Formally, a matched dataset can be defined as:

$$M = \{(i,j) \mid i \in \{1, \ldots, n\}, j \in \{1, \ldots, m\}, Z_i = 1, Z_j = 0, \text{ and } X_i \approx X_j\}.$$

In this definition, $X_i \approx X_j$ indicates that the covariates of the treated unit i and the control unit j are sufficiently similar based on some predefined measure of similarity (e.g., propensity scores, Euclidean distance, Mahalanobis distance, etc.).

Algorithm 1: k-Fold Cross-Validation to choose ε

Input: Dataset D, Matched dataset M, Number of folds k, grid of hyperparameters Λ, functional distance d

Randomly divide D into k subsets $D_1, D_2, \ldots, D_k$;

for *each fold* f *out of* k **do**

  Initialize training set E with all subsets except D_f;

  Initialize validation set V with subset D_f;

  Split M taking $S = \{i \in V : (i, j) \in M\}$;

  for $\varepsilon \in \Lambda$ **do**

    Compute $\hat{F}^{\varepsilon}_{\langle 0|1 \rangle}$ using E;

    Build $\tilde{F}^{\varepsilon}$ using $(\hat{F}^{\varepsilon}_{\langle 0|1 \rangle}(T^*_k), \Delta_k), \quad k \in S$

    Compute $d^{\varepsilon} = d(\tilde{F}^{\varepsilon}, \mathrm{id})$

  end

end

Average d^{ε} across all k folds;

In essence, by combining the strengths of both causal survival embeddings and the matched dataset, we are optimizing the model's performance within the constraints of the available data. This approach recognizes the theoretical sophistication of the causal survival embedding method while acknowledging the need to tailor it to the unique features of a particular dataset through parameter selection. This adds a practical dimension by fine-tuning the model to the specific evidence characteristics.

7 Numerical experiments: setup details

Embeddings estimator consistency

The underlying model for the simulation case study is

$$\log \tilde{T}^0 = X_1^0 + X_2^0 + \varepsilon$$
$$\log C^0 = X_1^0 + X_2^0 + \varepsilon'$$
$$\log \tilde{T}^1 = 2 + X_1^1 + X_2^1 + \omega$$
$$\log C^1 = 2 + X_1^1 + X_2^1 + \omega'$$

X_1^0 and X_2^0 are independent $\mathcal{N}(0,1)$ random variables while X_1^1 and X_2^1 are also independent unit variance normal but X_1^1 has mean 0.5. ε and ε' are $\mathcal{N}(c^0,1)$ and $\mathcal{N}(0,1)$ respectively with $c^0 > 0$ controlling the amount of censoring (the bigger c^0, the more censoring in the control arm). Analogously, ω and ω' are $\mathcal{N}(c^1,1)$ and $\mathcal{N}(0,1)$ respectively. We have set $c^0 = 0.2$ and $c^1 = 0.1$ in order to keep an incomplete information percentage of approximately 75% in both arms through all $B = 100$ simulation runs. We replicate the experiment for four different sample sizes $n = 100, 200, 300, 500$. We equip both the covariates and response spaces with Gaussian kernel $k(y, y') = \exp\left(-\left\|y - y'\right\|_2^2 / 2\sigma^2\right)$. The bandwidth parameter σ is chosen via the median heuristic: $\sigma^2 = \text{median}\left\{\left\|y_i - y_j\right\|_2^2 : i \neq j\right\} / 2$.

Survival functions estimator consistency

We present the details of the simulation setup used to generate synthetic data for our counterfactual analysis using causal survival embeddings. Two-dimensional covariates are generated as $X^0 \sim \mathcal{N}(0, \sigma^2 I)$ and $X^1 \sim \mathcal{N}(b, \sigma^2 I)$
Here, σ represents the standard deviation of the covariates, and b is a vector representing the shift applied to the treated group covariates.

The expected event rate for the control counterfactuals, μ_0, is calculated based on a quadratic function of the covariates, with coefficients represented by β_0: $\mu_{0,i} = (\beta_0' X_i)^2$ The expected event rate for the treatment counterfactuals, μ_1, is calculated based on a quadratic function of the covariates, with coefficients represented by β_1: $\mu_{1,i} = (\beta_1' X_i)^2$ Event times are generated for both counterfactuals using exponential and chi-squared distributions. For the control potential outcomes:

$$\tilde{T}_i^0 \sim \text{Exponential}\left(\frac{1}{\mu_{0,i}+1}\right)$$
$$C_i^0 \sim \chi^2(\mu_{0,i}+\delta_0)$$

For the treated potential outcomes:

$$\tilde{T}_i^1 - \xi \sim \text{Exponential}\left(\frac{1}{\mu_{1,i}+1}\right)$$
$$C_i^1 - \xi \sim \chi^2(\mu_{1,i}+\delta_1)$$

δ_0 and δ_1 are hyperparameters that tune the censoring percentage and ξ contributes to the distributional effect on the treated, complemented by how different β_0 and β_1 are.

Given this setting, Case I is simulated with the following configuration: $\beta_0 = (1,0), \quad \beta_1 = (0,-2), \quad b = (0,0), \quad \xi = 1$ and Case II, with $\beta_0 = (1,0), \quad \beta_1 = \beta_0, \quad b = (2,0), \quad \xi = 0$.

In our simulations, we evaluate the performance of our estimator using an equidistant grid of time points. At each of these time points, we calculate the squared pointwise difference between our estimated counterfactual survival function and the empirical version of $S_{\tilde{T}^0|Z=1}$. This empirical survival function is known precisely because of our controlled simulation setup, where we have access to both potential outcomes under both treatment indicators.

By computing these squared differences at each time point and then averaging them over the entire grid, we obtain a comprehensive measure of how closely our estimator aligns with the empirically observed survival function.

8 Description and naive analysis of SPRINT data

We conducted our analysis among 2269 participants older than 75 years old (as the stratification performed in Stensrud & Strohmaier (2017)) who had non-missing values for the covariates. Our response variable `T_PRIMARY` is observed time-to-primary outcome in days, which is a CVD composite endpoint of myocardial infarction, stroke, acute coronary syndrome, acute decompensated heart failure (ADHF), and CVD death. Composite outcomes are postulated to enhance the evaluation of treatment effects on infrequent outcomes, such as mortality in smaller trials, and serve as a convenient means of representing a broader spectrum of beneficial effects resulting from an intervention (Cordoba et al. 2010). Even considering several events to build the primary endpoint, the percentage of uncensored observations is 11 % and 7 % in the control and treatment arms respectively. These high incomplete information percentages render the consideration of censoring mandatory, consitituting a strong motivation factor for the development of our new estimator.

We stratify data into two groups: one with DBP $\leq$ 60 mmHg one year after randomisation (encoded `DBP60=0`) and a group with $>$ 60 mmHg one year after randomisation (encoded `DBP60=1`). Then we regress the primary endpoint against the newly created indicator variable using vanilla Cox PH:

```
> library('survival')

> primary=Surv(t,delta)
```

```
> coxdbp60 <- coxph(primary ~ DBP60)
> summary(coxdbp60)

coxph(formula = primary ~ DBP60)

  n= 2269, number of events= 210

         coef exp(coef) se(coef)     z Pr(>|z|)
DBP601 0.2823    1.3262   0.1475 1.914   0.0556 .
---
Signif. codes:  0 ‘***’ 0.001 ‘**’ 0.01 ‘*’ 0.05 ‘.’ 0.1 ‘ ’ 1

       exp(coef) exp(-coef) lower .95 upper .95
DBP601     1.326      0.754    0.9933     1.771

Concordance= 0.529  (se = 0.017 )
Likelihood ratio test= 3.54  on 1 df,   p=0.06
Wald test            = 3.66  on 1 df,   p=0.06
Score (logrank) test = 3.69  on 1 df,   p=0.05
```

9 Proofs of auxiliary results

Proof of Lemma 1 (in main text)

We have for an arbitrary $G \in \mathcal{F}$

$$\hat{R}_{\varepsilon,n}(\hat{F}+G) = \frac{1}{n}\sum_{i=1}^{n} W_i \left\| h_i - \hat{F}\left(X_i\right) - G\left(X_i\right) \right\|_{\mathcal{H}}^2 + \varepsilon \|\hat{F}+G\|_{\mathcal{F}}^2 =$$

$$\frac{1}{n}\sum_{i=1}^{n} W_i \left(\left\| h_i - \hat{F}\left(X_i\right) \right\|_{\mathcal{H}}^2 + \|G\left(X_i\right)\|_{\mathcal{H}}^2 - 2\langle h_i - \hat{F}(X_i), G(X_i)\rangle_{\mathcal{H}} \right) + \varepsilon \left(\|\hat{F}\|_{\mathcal{F}}^2 + \|G\|_{\mathcal{F}}^2 - 2\langle \hat{F}, G\rangle_{\mathcal{F}} \right) =$$

$$\hat{R}_{\varepsilon,n}(\hat{F}) + \frac{1}{n}\sum_{i=1}^{n} W_i \left(\|G\left(X_i\right)\|_{\mathcal{H}}^2 - 2\langle h_i - \hat{F}(X_i), G(X_i)\rangle_{\mathcal{H}} \right) + \varepsilon \left(\|G\|_{\mathcal{F}}^2 + 2\langle \hat{F}, G\rangle_{\mathcal{F}} \right)$$

Assuming that $\hat{F}$ is a minimizer implies that $\hat{R}_{\varepsilon,n}(\hat{F}) \leq \hat{R}_{\varepsilon,n}(\hat{F}+G)$ and therefore it is necessary that for all $G \in \mathcal{F}$

$$\frac{1}{n}\sum_{i=1}^{n} W_i \langle h_i - \hat{F}(X_i), G(X_i)\rangle_{\mathcal{H}} = \varepsilon \langle \hat{F}, G\rangle_{\mathcal{F}}$$

Let us try the solution $\hat{F} = \sum_{i=1}^{n} \Gamma\left(\cdot, X_i\right)\left(c_i\right) \in \mathcal{F}$ and we use the properties of Γ to develop the inner product

$$\langle \hat{F}, G\rangle_{\mathcal{F}} = \langle \sum_{i=1}^{n} \Gamma\left(\cdot, X_i\right)\left(c_i\right), G\rangle_{\mathcal{F}} = \sum_{i=1}^{n} \langle c_i, G(X_i)\rangle_{\mathcal{H}}$$

So,

$$\frac{1}{n}\sum_{i=1}^{n} W_i \langle h_i - \hat{F}(X_i), G(X_i)\rangle_{\mathcal{H}} = \varepsilon \sum_{i=1}^{n} \langle c_i, G(X_i)\rangle_{\mathcal{H}}$$

Therefore

$$\sum_{i=1}^{n} \left(W_i \langle h_i - \hat{F}(X_i), G(X_i)\rangle_{\mathcal{H}} - n\varepsilon \langle c_i, G(X_i)\rangle_{\mathcal{H}} \right) = 0$$

Now we use again the expression $\hat{F} = \sum_{i=1}^{n} \Gamma(\cdot, X_i)(c_i)$ to rewrite

$$\langle \hat{F}(X_i), G(X_i) \rangle_{\mathcal{H}} = \sum_{j=1}^{n} \langle \Gamma(X_i, X_j)(c_j), G(X_i) \rangle_{\mathcal{H}}$$

For the previous identity to be true for all $G \in \mathcal{F}$, it is sufficient that for $1 \leq i \leq n$ the following holds

$$W_i(h_i - \sum_{j=1}^{n} \Gamma(X_i, X_j)(c_j)) - n\varepsilon c_i = 0$$

that can be written as

$$W_i h_i = \sum_{j=1}^{n} W_i \Gamma(X_i, X_j)(c_j) + n\varepsilon c_i \delta_{ij}$$

Proof of Lemma 2

Define $g := \left(\widehat{\mathcal{C}^*}_{XX} + \varepsilon I\right)^{-1} \hat{\mu}_{X_1}$.
Since $\hat{\mu}_{X_1} = \left(\widehat{\mathcal{C}^*}_{XX} + \varepsilon I\right) g = \frac{1}{n} \sum_{j=1}^{n} W_j k(\cdot, X_j) g(X_j) + \varepsilon g$,
we have $\hat{\mu}_{X_1}(X_l) = \frac{1}{n} \sum_{j=1}^{n} W_j k(X_l, X_j) g(X_j) + \varepsilon g(X_l) = \frac{1}{n}(KW\boldsymbol{g})_l + \varepsilon \boldsymbol{g}_l$ for all $l = 1, \ldots, n$, where $K \in \mathbb{R}^{n \times n}$ with $K_{ij} = k(X_i, X_j)$ and $\boldsymbol{g} = (g(X_1), \ldots, g(X_n))^\top \in \mathbb{R}^n$. Therefore $\boldsymbol{\mu} = \frac{1}{n}(KW + n\varepsilon I)\boldsymbol{g}$, where $\boldsymbol{\mu} := (\hat{\mu}_{X_1}(X_1), \ldots, \hat{\mu}_{X_1}(X_n))^\top = \widetilde{K} 1_m$, where $1_m = (1/m, \ldots, 1/m)^\top$ and $\widetilde{K} \in \mathbb{R}^{n \times m}$ with $\widetilde{K}_{ij} = k\left(X_i, X_j^1\right)$. Thus $\boldsymbol{g} = n(KW + n\varepsilon I)^{-1}\boldsymbol{\mu}$. Lastly, we use the definition of $\widehat{\mathcal{C}^*}_{TX}$ to express $\hat{\mu}_{\langle 0|1 \rangle} = \frac{1}{n} \sum_{i=1}^{n} W_i \ell(\cdot, Y_i) g(X_i) = \sum_{i=1}^{n} W_i \beta_i \ell(\cdot, Y_i)$, where $\beta = (\beta_1, \ldots, \beta_n)^\top = n^{-1}\boldsymbol{g} = (KW + n\varepsilon I)^{-1}\boldsymbol{\mu}$, which is the original expression of $\hat{\mu}_{T\langle 0|1 \rangle}$.

Proof of Lemma 2

$$\left\|\frac{1}{n}\left(\sum_{i=1}^{n}W_iK_iL_i-\frac{2}{n}\left(\sum_{i=1}^{n}W_iK_i\right)\left(\sum_{i=1}^{n}W_iL_i\right)+\frac{1}{n^2}\left(\sum_{i=1}^{n}W_iK_i\right)\left(\sum_{i=1}^{n}W_iL_i\right)\left(\sum_{i=1}^{n}W_i\right)\right)-E[K(X^0)L(T^0)]\right\|_{\mathcal{G}\otimes\mathcal{H}}$$
$$=\left\|\frac{1}{n}\sum_{i=1}^{n}W_iK_iL_i-2\left(\frac{1}{n}\sum_{i=1}^{n}W_iK_i\right)\left(\frac{1}{n}\sum_{i=1}^{n}W_iL_i\right)+\left(\frac{1}{n}\sum_{i=1}^{n}W_iK_i\right)\left(\frac{1}{n}\sum_{i=1}^{n}W_iL_i\right)\left(\frac{1}{n}\sum_{i=1}^{n}W_i\right)-E[K(X^0)L(T^0)]\right\|_{\mathcal{G}\otimes\mathcal{H}}$$
$$=\left\|\frac{1}{n}\sum_{i=1}^{n}W_iK_iL_i-E[K(X^0)L(T^0)]-\left(2-\frac{1}{n}\sum_{i=1}^{n}W_i\right)\left(\frac{1}{n}\sum_{i=1}^{n}W_iK_i\right)\left(\frac{1}{n}\sum_{i=1}^{n}W_iL_i\right)\right\|_{\mathcal{G}\otimes\mathcal{H}}$$
$$\leq\left\|\frac{1}{n}\sum_{i=1}^{n}W_iK_iL_i-E[K(X^0)L(T^0)]\right\|_{\mathcal{G}\otimes\mathcal{H}}+\left|2-\frac{1}{n}\sum_{i=1}^{n}W_i\right|\left\|\left(\frac{1}{n}\sum_{i=1}^{n}W_iK_i\right)\left(\frac{1}{n}\sum_{i=1}^{n}W_iL_i\right)\right\|_{\mathcal{G}\otimes\mathcal{H}}$$
$$\leq\left\|\frac{1}{n}\sum_{i=1}^{n}W_iK_iL_i-E[K(X^0)L(T^0)]\right\|_{\mathcal{G}\otimes\mathcal{H}}+\left|2-\frac{1}{n}\sum_{i=1}^{n}W_i\right|\left\|\left(\frac{1}{n}\sum_{i=1}^{n}W_iK_i\right)\right\|_{\mathcal{G}}\left\|\left(\frac{1}{n}\sum_{i=1}^{n}W_iL_i\right)\right\|_{\mathcal{H}}$$

Proof of Corollary 1

By Triangle's Inequality,

$$\begin{aligned}&\|\widehat{\mathcal{C}}_{TX}\left(\widehat{\mathcal{C}}_{XX}+\varepsilon_nI\right)^{-1}\hat{\mu}_{X_1}-\mu_{T\langle 0|1\rangle}\|_{\mathcal{H}}\\&\quad\leq\left\|\widehat{\mathcal{C}}_{TX}\left(\widehat{\mathcal{C}}_{XX}+\varepsilon_nI\right)^{-1}\hat{\mu}_{X_1}-\mathcal{C}_{TX}\left(\mathcal{C}_{XX}+\varepsilon_nI\right)^{-1}\mu_{X_1}\right\|_{\mathcal{H}}\quad\text{(Stochastic error)}\\&\qquad+\left\|\mathcal{C}_{TX}\left(\mathcal{C}_{XX}+\varepsilon_nI\right)^{-1}\mu_{X_1}-\mu_{T\langle 0|1\rangle}\right\|_{\mathcal{H}}\quad\text{(Approximation error)}\end{aligned}$$

Proof of Corollary 2

The proof of Theorem 2 has been written with $\alpha = 0$ (we can assume so thanks to assumption iii.)). The proof for $\alpha > 0$ is straightforward using Lemma 24 in Muandet et al. (2017). In this case, the rate of the stochastic error is $O_p\left(n^{-1/2}\varepsilon_n^{\min(-1+\alpha,-1/2)}\right)$. The proof is completed by showing that the rate for the approximation error is $O\left(\varepsilon_n^{(\alpha+\beta)/2}\right)$ (see E.3 in Muandet et al. (2021)).

References

Aalen, O. O., Cook, R. J. & Røysland, K. (2015), 'Does cox analysis of a randomized survival study yield a causal treatment effect?', *Lifetime data analysis* **21**(4), 579–593.

Abadie, A. (2002), 'Bootstrap tests for distributional treatment effects in instrumental variable models', *Journal of the American Statistical Association* **97**(457), 284–292.
URL: *https://doi.org/10.1198/016214502753479419*

Andersen, P. K., Borgan, O., Gill, R. D. & Keiding, N. (2012), *Statistical models based on counting processes*, Springer Science & Business Media.

Baker, C. R. (1973), 'Joint measures and cross-covariance operators', *Transactions of the American Mathematical Society* **186**, 273–289.

Berlinet, A. & Thomas-Agnan, C. (2011), *Reproducing kernel Hilbert spaces in probability and statistics*, Springer Science & Business Media.

Blinder, A. S. (1973), 'Wage discrimination: Reduced form and structural estimates', *The Journal of Human Resources* **8**(4), 436–455.
URL: *http://www.jstor.org/stable/144855*

Carmeli, C., De Vito, E. & Toigo, A. (2006), 'Vector valued reproducing kernel hilbert spaces of integrable functions and mercer theorem', *Analysis and Applications* **4**(04), 377–408.

Chernozhukov, V., Fernández-Val, I. & Melly, B. (2013), 'Inference on counterfactual distributions', *Econometrica* **81**(6), 2205–2268.
URL: *https://onlinelibrary.wiley.com/doi/abs/10.3982/ECTA10582*

Cordoba, G., Schwartz, L., Woloshin, S., Bae, H. & Gøtzsche, P. C. (2010), 'Definition, reporting, and interpretation of composite outcomes in clinical trials: systematic review', *Bmj* **341**.

Cox, D. R. (1972), 'Regression models and life-tables', *Journal of the Royal Statistical Society: Series B (Methodological)* **34**(2), 187–202.

Fernández, T. & Gretton, A. (2019), A maximum-mean-discrepancy goodness-of-fit test for censored data, *in* 'The 22nd International Conference on Artificial Intelligence and Statistics', PMLR, pp. 2966–2975.

Fleming, T. R. & Harrington, D. P. (2011), *Counting processes and survival analysis*, John Wiley & Sons.

Fukumizu, K., Bach, F. R. & Gretton, A. (2007), 'Statistical consistency of kernel canonical correlation analysis', *Journal of Machine Learning Research* **8**(14), 361–383.
URL: *http://jmlr.org/papers/v8/fukumizu07a.html*

Fukumizu, K., Song, L. & Gretton, A. (2013), 'Kernel bayes' rule: Bayesian inference with positive definite kernels', *Journal of Machine Learning Research* **14**(118), 3753–3783.
URL: *http://jmlr.org/papers/v14/fukumizu13a.html*

Gao, Z., Hastie, T. & Tibshirani, R. (2021), 'Assessment of heterogeneous treatment effect estimation accuracy via matching', *Statistics in medicine* **40**(17), 3990–4013.

Hernán, M. A. (2010), 'The hazards of hazard ratios', *Epidemiology (Cambridge, Mass.)* **21**(1), 13.

Horvitz, D. G. & Thompson, D. J. (1952), 'A generalization of sampling without replacement from a finite universe', *Journal of the American statistical Association* **47**(260), 663–685.

Imbens, G. W. (2004), 'Nonparametric estimation of average treatment effects under exogeneity: A review', *Review of Economics and statistics* **86**(1), 4–29.

Imbens, G. W. & Rubin, D. B. (1997), 'Estimating outcome distributions for compliers in instrumental variables models', *The Review of Economic Studies* **64**(4), 555–574.

Juhn, C., Murphy, K. M. & Pierce, B. (1993), 'Wage inequality and the rise in returns to skill', *Journal of political Economy* **101**(3), 410–442.

Kanagawa, M. & Fukumizu, K. (2014), Recovering Distributions from Gaussian RKHS Embeddings, *in* S. Kaski & J. Corander, eds, 'Proceedings of the Seventeenth International Conference on Artificial Intelligence and Statistics', Vol. 33 of *Proceedings of Machine Learning Research*, PMLR, Reykjavik, Iceland, pp. 457–465.
URL: *https://proceedings.mlr.press/v33/kanagawa14.html*

Kaplan, E. L. & Meier, P. (1958), 'Nonparametric estimation from incomplete observations', *Journal of the American statistical association* **53**(282), 457–481.

Kosorok, M. R. (2008), *Introduction to empirical processes and semiparametric inference.*, Springer.

Ledoux, M. & Talagrand, M. (1991), *Probability in Banach Spaces: isoperimetry and processes*, Vol. 23, Springer Science & Business Media.

Lee, B. K., Lessler, J. & Stuart, E. A. (2010), 'Improving propensity score weighting using machine learning', *Statistics in medicine* **29**(3), 337–346.

Ma, X. & Wang, J. (2020), 'Robust inference using inverse probability weighting', *Journal of the American Statistical Association* **115**(532), 1851–1860.
URL: *https://doi.org/10.1080/01621459.2019.1660173*

Martinussen, T. (2022), 'Causality and the cox regression model', *Annual Review of Statistics and Its Application* **9**, 249–259.

Micchelli, C. A. & Pontil, M. (2005), 'On learning vector-valued functions', *Neural computation* **17**(1), 177–204.

Muandet, K., Fukumizu, K., Sriperumbudur, B. & Schölkopf, B. (2017), 'Kernel mean embedding of distributions: A review and beyond', *Foundations and Trends® in Machine Learning* **10**(1-2), 1–141.
URL: *https://arxiv.org/pdf/1605.09522.pdf*

Muandet, K., Kanagawa, M., Saengkyongam, S. & Marukatat, S. (2021), 'Counterfactual mean embeddings', *Journal of Machine Learning Research* **22**(162), 1–71.
URL: *http://jmlr.org/papers/v22/20-185.html*

Neyman, J. (1923), 'Sur les applications de la theorie des probabilites aux experiences agricoles: Essai des principes (in polish). english translation by dm dabrowska and tp speed (1990)', *Statistical Science* **5**, 465–480.

Oaxaca, R. (1973), 'Male-female wage differentials in urban labor markets', *International Economic Review* **14**(3), 693–709.
URL: *http://www.jstor.org/stable/2525981*

Park, J., Shalit, U., Schölkopf, B. & Muandet, K. (2021), Conditional distributional treat-

ment effect with kernel conditional mean embeddings and u-statistic regression, *in* 'International Conference on Machine Learning', PMLR, pp. 8401–8412.

Pearl, J. et al. (2000), 'Models, reasoning and inference', *Cambridge, UK: Cambridge University Press* **19**(2).

Reed, M. & Simon, B. (1980), *Methods of modern mathematical physics. vol. 1. Functional analysis*, Academic New York.

Robins, J. & Greenland, S. (1989), 'The probability of causation under a stochastic model for individual risk', *Biometrics* pp. 1125–1138.

Rosenbaum, P. R. & Rubin, D. B. (1983), 'The central role of the propensity score in observational studies for causal effects', *Biometrika* **70**(1), 41–55.

Rubin, D. B. (1974), 'Estimating causal effects of treatments in randomized and nonrandomized studies.', *Journal of educational Psychology* **66**(5), 688.

Song, L., Fukumizu, K. & Gretton, A. (2013), 'Kernel embeddings of conditional distributions: A unified kernel framework for nonparametric inference in graphical models', *IEEE Signal Processing Magazine* **30**(4), 98–111.

Stensrud, M. J., Aalen, J. M., Aalen, O. O. & Valberg, M. (2018), 'Limitations of hazard ratios in clinical trials', *European Heart Journal* **40**(17), 1378–1383.
URL: *https://doi.org/10.1093/eurheartj/ehy770*

Stensrud, M. J. & Strohmaier, S. (2017), 'Diastolic hypotension due to intensive blood pressure therapy: Is it harmful?', *Atherosclerosis* **265**, 29–34.
URL: *https://www.sciencedirect.com/science/article/pii/S0021915017311942*

Stock, J. H. (1989), 'Nonparametric policy analysis', *Journal of the American Statistical Association* **84**(406), 567–575.

Stute, W. (1993), 'Consistent estimation under random censorship when covariables are present', *Journal of Multivariate Analysis* **45**(1), 89–103.

Van der Vaart, A. W. (2000), *Asymptotic statistics*, Vol. 3, Cambridge university press.
